# Use of comorbidity indices in patients with any cancer, breast cancer, and human epidermal growth factor receptor-2-positive breast cancer: A systematic review

**DOI:** 10.1371/journal.pone.0252925

**Published:** 2021-06-18

**Authors:** Maribel Salas, Mackenzie Henderson, Meera Sundararajan, Nora Tu, Zahidul Islam, Mina Ebeid, Laura Horne

**Affiliations:** 1 Global Epidemiology, Clinical Safety and Pharmacovigilance, Daiichi Sankyo Inc., Basking Ridge, NJ, United States of America; 2 Center for Clinical Epidemiology and Biostatistics (CCEB)/Center for Pharmacoepidemiology Research and Training (CPeRT), University of Pennsylvania Perelman School of Medicine, Philadelphia, PA, United States of America; 3 Rutgers Institute for Pharmaceutical Industry Fellowships, Rutgers University, New Brunswick, NJ, United States of America; 4 Clinical Safety and Pharmacovigilance, Daiichi Sankyo Inc., Basking Ridge, NJ, United States of America; UT MD Anderson Cancer Center, UNITED STATES

## Abstract

**Objective:**

To identify comorbidity indices that have been validated in cancer populations, with a focus on breast cancer and human epidermal growth factor receptor-2-positive (HER2+) breast cancer.

**Study design and setting:**

A systematic review of the literature on the use of comorbidity indices in any cancer, breast cancer, and HER2+ breast cancer using Ovid and PubMed.

**Results:**

The final data set comprised 252 articles (252 any cancer, 39 breast cancer, 7 HER2+ breast cancer). The most common cancers assessed were hematologic and breast, and the most common comorbidity index used was the Charlson Comorbidity Index (CCI) or a CCI derivative. Most validity testing of comorbidity indices used predictive validity based on survival outcomes. Hazard ratios for survival outcomes generally found that a higher comorbidity burden (measured by CCI) increased mortality risk in patients with breast cancer. All breast-cancer studies that validated comorbidity indices used CCI-based indices. Only one article validated a comorbidity index in HER2+ breast cancer.

**Conclusion:**

CCI-based indices are the most appropriate indices to use in the general breast-cancer population. There is insufficient validation of any comorbidity index in HER2+ breast cancer to provide a recommendation, indicating a future need to validate these instruments in this population.

## 1. Introduction

The presence of comorbidities in patients with cancer may lead to a delayed cancer diagnosis, suboptimal treatment, and an increased risk of postoperative complications and mortality [1]. A review of 2,500 articles reported 5-year mortality hazard ratios (HRs) ranging from 1.1–5.8 for patients with cancer with comorbidities compared to those without comorbidities [1]. Current estimates indicate that comorbidities are common, with 20–35% of patients with breast cancer having at least one [1]. In patients with human epidermal growth factor receptor-2-positive (HER2+) breast cancer, there is evidence that comorbidities affect HER2+-targeted treatment completion [2] and treatment decisions. Furthermore, treatment with strict adherence to guidelines is significantly associated with improved outcomes in patients with HER2+ breast cancer [3]. Therefore, the presence of comorbidities has the potential to influence treatment decisions and treatment outcomes in patients with breast cancer and HER2+ breast cancer.

There are two ways in which comorbidities are typically measured and included in epidemiological research. The first is to simply use presence/absence of specific comorbidities as covariates in statistical analyses. The second is using comorbidity data to create a comorbidity index that weights each comorbidity, and the sum of the weights generates a numeric score [4]. Examples include the Charlson Comorbidity Index (CCI), Adult Comorbidity Evaluation-27 (ACE-27), and Elixhauser Comorbidity Measure (ECM), which are commonly used general comorbidity indices [5–7]. Other comorbidity indices are available, including an extension of the original CCI, which combines age and comorbidity [8], and disease-specific indices such as the Prostate Cancer Comorbidity Index (PCCI) [9] and the Hematopoietic Cell Transplantation Comorbidity Index (HCT-CI) [10]. Comorbidity indices are generally designed for use in an observational setting, e.g., as research tools in retrospective analyses of patient data from administrative databases [4, 11]. Comorbidity indices such as the CCI, ACE-27, and Cumulative Illness Rating Scale-Geriatric (CIRS-G) have also been used in clinical practice, particularly in assessing elderly patients where they are combined with assessment of functional and global status [12].

For a comorbidity index to be used in clinical practice, it needs to undergo rigorous validation and reliability testing [13, 14]. The CCI has been validated in several cancer types, including head and neck, and non-small-cell lung cancer [15, 16]. A search of the literature did not identify any robust reviews or meta-analyses evaluating the CCI or other comorbidity indices in patients with breast cancer.

This systematic review was designed to identify in published literature validated comorbidity indices in patients with any type of cancer (1), breast cancer (2), and HER2+ breast cancer (3). Secondary objectives were to evaluate the methodology of validation studies, and identify appropriate comorbidity indices for future use in clinical practice or research of patients with any cancer, breast cancer, or HER2+ breast cancer.

## 2. Methods

### 2.1 Study design

A systematic review of the literature on the use of comorbidity indices in any cancer, breast cancer, and HER2+ breast cancer was undertaken. Articles published between January 1, 2010 and February 5, 2020 in humans (English language) were evaluated. Searches using pre-defined search terms were conducted in Ovid (Medline, Embase, Biosis) and PubMed. The search strategy included an initial search for references on “any cancer”, followed by specific searches for “breast cancer” and “HER2+ breast cancer” references (for the full search strategy, see S1 Table).

### 2.2 Eligibility criteria

Eligible studies included interventional (e.g., clinical trials) and non-interventional research design studies (e.g., cohort studies, case-control studies, and cross-sectional studies) and specifically included a comorbidity index that was used to evaluate comorbidities of the study participants. To ensure articles presenting validated comorbidity indices were found, initial search terms included the word “validation” and English variations of this word. Reviews, meta-analyses, case reports, case studies, case series, pre-clinical studies, non-human studies, editorials, commentaries, letters to editors, and meeting/conference abstracts were excluded (although meta-analysis and review articles were reviewed for potentially relevant references).

The “any cancer” review included articles evaluating patients with a diagnosis of any type of cancer affecting any organ in the body. A single study could include several types of cancer. The “breast cancer” review included articles evaluating patients with a diagnosis of any type of breast cancer, including all stages (1–4) and subtypes (HER2+ and HER2 negative [HER2-], hormone receptor-positive [HR+], estrogen receptor-positive/progesterone receptor-positive HR+ [ER+/PR+] and hormone receptor-negative [HR-]; estrogen receptor-negative/progesterone receptor-negative HR- [ER-/PR-]; triple negative; and others). The “HER2+ breast cancer” review included patients with a diagnosis of HER2+ breast cancer, including all stages (1–4), HR+ (ER+/PR+) and HR- (ER-/PR-), and others.

### 2.3 Screening articles

Titles and abstracts of all articles identified from the literature searches were exported into Microsoft^®^ Excel for eligibility screening and data management. Two levels of article selection were completed. In level-1 screening, titles and abstracts of articles were reviewed. Full texts of articles chosen in level-1 screening were reviewed in level-2 screening. Two researchers completed article selection independently; any uncertainty over inclusion of an article was resolved by discussion and agreement between the two researchers. Relevant references in reviews and meta-analyses found in the literature search were also reviewed for inclusion. The results of the article screening process were documented according to the Preferred Reporting Items for Systemic Reviews and Meta-analysis (PRISMA).

The methods sections of included articles were evaluated to assess methodology and risk of bias according to the Newcastle-Ottawa Scale for cohort and case-control research designs and the Cochrane criteria for assessing bias in interventional research designs [17, 18].

### 2.4 Data extraction

Data were manually extracted from each selected article by a researcher and imported into Microsoft^®^ Excel for data extraction and review. Study characteristics extracted included: study/trial design; country/setting of research; year(s) of research; specific disease state(s) evaluated; objective(s) of study/trial; demographic information of the study population; specific tumor information for included patients; and sample size. Comorbidity index characteristics extracted included: name of comorbidity index used in the research; type of validation (when applicable), including reliability, content validity, construct validity, criterion validity, responsiveness, and interpretability; and validation results of comorbidity index.

Any comorbidity index including the words Charlson Comorbidity Index (CCI) in its title was defined as a CCI derivative.

### 2.5 Outcome

The outcome of interest was the validity and/or reliability results for specific comorbidity indices. Specific comorbidity scores of patients reported in individual studies are not included in this systematic review. Quantitative and qualitative validity and/or reliability results of comorbidity indices were extracted and reported.

An independent researcher performed quality control to ensure accurateness and thoroughness of data extracted from the articles that were included in the systematic review. Agreement between the researcher who performed data extraction and the researcher who performed quality control on the extracted data (defined as the number of fields the researchers agreed on divided by the total number of fields, multiplied by 100%) was greater than 90% overall.

### 2.6 Statistical methods

No inferential statistical analysis was conducted. Descriptive statistics, including frequencies, were used to provide a summary of the included articles.

## 3. Results

**Fig 1:**
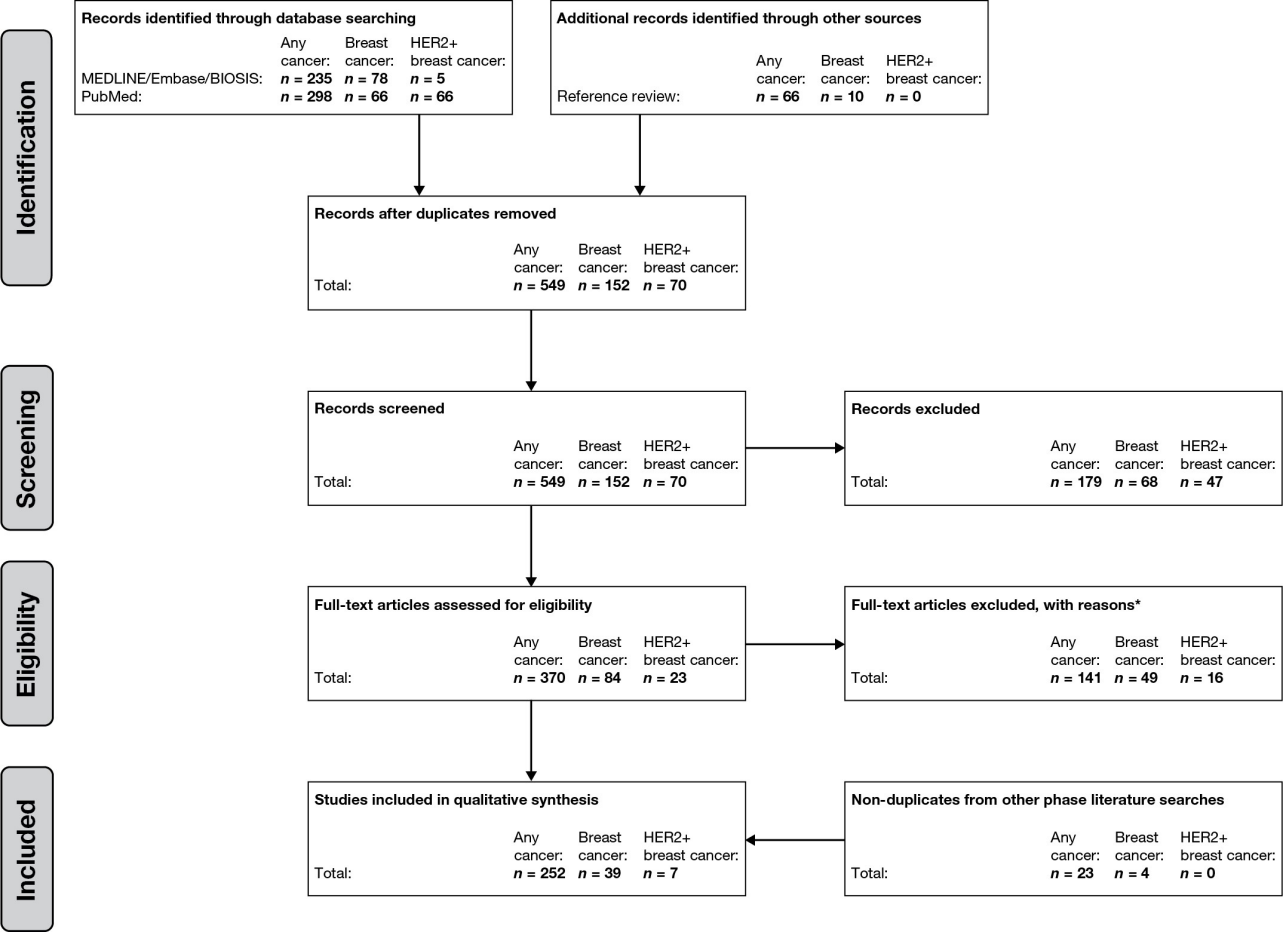
PRISMA flow diagram for “any cancer”, “breast cancer”, and “HER2+ breast cancer”. *Reasons for exclusion: Any cancer: 28 abstract terminal documents; 1 guideline; 2 expert panel; 1 focus on cancer screening; 1 non-English; 72 no comorbidity index used; 5 focus not on comorbidity; 13 not focused on patients with cancer or patient population not well-defined; 16 not primary literature (review); 1 date range out of scope; 1 not available from publisher. Breast cancer: 43 no mention of comorbidity index; 2 focus on treatment decision; 2 non-English; 2 not primary literature. HER2+ breast cancer: 13 no mention of comorbidity index; 3 not focused on HER2+ breast cancer. *HER2+* human epidermal growth factor receptor-2-positive, *PRISMA* Preferred Reporting Items for Systemic Reviews and Meta-analysis.

We identified 549 articles for “any cancer”, 152 for “breast cancer”, and 70 for “HER2+ breast cancer”. After abstract and full-text screening, 252, 39, and 7 articles, respectively, were included in the final data set (Fig 1).

A range of cancers was evaluated in the “any cancer” category. The most common (> 25 articles) were hematologic, breast, head and neck, lung, colorectal, and urologic cancers (Table 1).

**Table 1:** Types of cancer evaluated in articles included in this review.

Cancer	Any cancer	Breast cancer
	(*N* = 252)	(*N* = 39)
Hematologic*	48	–
Breast	47	39
Unspecified breast	–	27
HER2+ only	–	7
HER2-/HR+ only	–	1
HR+ only	–	2
HR- only	–	1
Male only	–	1
Lung^†^	29	–
Head and neck^‡^	29	–
Colorectal^§^	28	–
Urologic/urinary tract	28	–
Prostate	23	–
Gynecologic^¶^	12	–
Renal/kidney	9	–
Gastrointestinal^|^	8	–
Liver**	6	–
Pancreatic	6	–
Central nervous system^††^	4	–
Bone	1	–
Skin	1	–
Others	15	–

Articles may have evaluated more than one cancer, so cell totals may not add up to column totals.

*Including adult T-cell leukemia/lymphoma, acute lymphoid/lymphoblastic leukemia, acute myeloid leukemia, B-cell lymphoma, chronic lymphocytic leukemia, chronic monomyelocytic leukemia, chronic myeloid/myelogenous leukemia, Hodgkin lymphoma, mixed phenotypic acute leukemia, multiple myeloma, myeloproliferative disorder/neoplasm, and non-Hodgkin lymphoma.

^†^Including mesothelioma, non-small-cell lung cancer, small-cell lung cancer, and unspecified.

^‡^Including esophageal, hypopharyngeal, laryngeal, maxillary, nasopharyngeal, oral, oropharyngeal, and unspecified.

^§^Including anal, colon, malignant colon obstruction, rectal, and unspecified.

^¶^Including endometrial, ovarian, uterine, and unspecified.

^|^Including gastric/stomach, upper gastrointestinal, and unspecified.

**Including hepatocellular carcinoma and unspecified.

^††^Including unspecified brain, glioblastoma, meningioma, and spinal.

*HER2+* human epidermal growth factor receptor-2-positive, *HR+* hormone receptor-positive, *HR-* hormone receptor-negative.

### 3.1 Comorbidity indices

A range of comorbidity indices were included in the articles. The most commonly used index was the CCI or a derivative, which was used in 174/252 (69.0%) “any cancer” articles, 33/39 (84.6%) “breast cancer” articles, and 5/7 (71.4%) “HER2+ breast cancer” articles. Derivatives of the CCI followed the original CCI index with one or more of the following alterations: incorporation of additional variables (e.g., age-adjusted CCI) or coding algorithms to identify variables in databases (e.g., Deyo-adapted CCI), or removal of variables (e.g., non-cancer CCI) [19–21]. In some cases, modifications of the CCI were unclear.

Following the CCI, the next most commonly used comorbidity indices were HCT-CI, which was used by 35/252 (13.9%) articles, and the ACE-27, which was used by 27/252 (10.7%) articles. The HCT-CI was particularly commonly used in articles that evaluated hematologic cancers (35/48 [72.9%] articles; Table 2).

**Table 2:** Comorbidity indices used in the articles included in this review.

Cancer	Any cancer	Breast cancer	HER2+ breast cancer
	(*N* = 252)	(*N* = 39)	(*N* = 7)
Charlson Comorbidity Index (CCI)	174	33	5
Original CCI	132	20	3
Charlson-Deyo Comorbidity Index	18	9	1
Age-Adjusted CCI	14	–	–
Klabunde Modified CCI*	4	2	1
Non-Cancer CCI	2	–	–
Modified CCI	2	1	–
Deyo and Romano adapted CCI	1	–	–
Hypertension Augmented CCI	1	1	–
Quan CCI	1	1	–
Short Form CCI	1	–	–
Revised CCI (Spanish)	1	–	–
Hematopoietic Cell Transplant Comorbidity Index	35	–	–
Adult Comorbidity Evaluation-27	27	3	1
Elixhauser Comorbidity Index	11	–	–
Cumulative Illness Rating Scale	7	–	–
Self-Administered Comorbidity Score	4	2	–
Other^†^	23	1	1

Articles may have evaluated more than one comorbidity index, so cell totals may not add up to column totals.

*Including the National Cancer Institute Comorbidity Index.

^†^Including the C3 Index, Colorectal Cancer Comorbidity Index, Comorbidity-EBMT (European Society for Blood and Bone Marrow Transplantation) Index, Freiburg Comorbidity Index, Head and Neck Comorbidity Index, Index of Coexistent Disease, Inpatient Bed-Day Comorbidity Index, Kaplan-Feinstein Comorbidity Index, Ovarian Cancer Comorbidity Index, Osaka Head and Neck Comorbidity Index, Prostate Cancer Comorbidity Index, Revised Myeloma Comorbidity Index, Rx-Risk/Rx-Risk-V Comorbidity Index, Simplified Comorbidity Score, Washington University Head and Neck Comorbidity Index.

*HER2+*, human epidermal growth factor receptor-2-positive.

### 3.2 Study designs

The vast majority of studies included were observational (246/252 [97.6%] “any cancer”, 38/39 [97.4%] “breast cancer”, and 7/7 [100%] “HER2+ breast cancer”). Articles describing interventional trial designs accounted for only 6 (2.4%) “any cancer” articles and 1 (2.6%) “breast cancer” article; no interventional studies of HER2+ breast cancer were identified in this review. Sample sizes varied greatly across studies. The majority of “any cancer” and “breast cancer” articles were from studies of > 500 patients (62.3% and 74.4%, respectively). The most common region where studies were conducted was North America (Table 3).

**Table 3:** Study characteristics of articles included in this review.

Characteristic	Any cancer	Breast cancer	HER2+ breast cancer
	(*N* = 252)	(*N* = 39)	(*N* = 7)
**Study design (number of articles)**			
Observational study (total)	246	38	7
Case control	2	–	–
Cohort	235	36	7
Cross-sectional	9	2	–
Interventional (total)	6	1	–
**Sample size (number of articles)**			
0–≤ 50	9	1	–
51–≤ 100	23	2	–
101–≤ 500	63	7	2
501–≤ 1,000	47	5	–
1,001–≤ 5,000	43	6	1
> 5,000	67	18	4
**Region (number of articles)**			
Asia*	32	3	–
Australia/New Zealand	7	1	1
Europe^†^	84	11	2
North America^‡^	121	23	4
South America^§^	2	–	–
Unclear	10	1	–

Some studies were performed in multiple regions, so cell totals may not add up to column totals.

*Including China, India, Israel, Japan, South Korea, Taiwan, and Turkey.

^†^Including Austria, Belgium, Denmark, Finland, France, Germany, Italy, Lithuania, The Netherlands, Norway, Spain, Sweden, and the United Kingdom.

^‡^Including Canada and the United States.

^§^Including Brazil.

*HER2+*, human epidermal growth factor receptor-2-positive.

### 3.3 Risk of bias analysis

The Newcastle-Ottawa Scale was used to assess the risk of bias in 235 cohort studies and two case-control studies. Most of the cohort studies were rated 5 (81/235 [34.5%]) or 4 stars (58/235 [24.7%]), and the case-control studies were rated 7 and 6 stars out of a possible 9 stars; a higher number indicated less risk of bias according to the scale. Most cohort studies (about 59.1%) scored either 4 or 5 out of 9 stars, indicating that there was at least a moderate risk of bias in the majority of the cohort studies included. All case-control studies (100%), scored at least 6 out of 9 stars, indicating that there was a lower risk of bias in the included case-control studies. The Cochrane criteria were used to assess bias in the six interventional studies. One study was classified as high risk, one as low risk, and four as unclear risk of bias. A rating of low or high risk of bias indicates that articles clearly stated their methodology and risk of bias was evaluable, whereas unclear risk (which categorized the majority of clinical trials included in this review) indicates significant portions of the methodology were not clearly stated and the overall risk of bias could not be determined.

### 3.4 Validation of comorbidity indices

#### 3.4.1 Predictive validity

**Fig 2:**
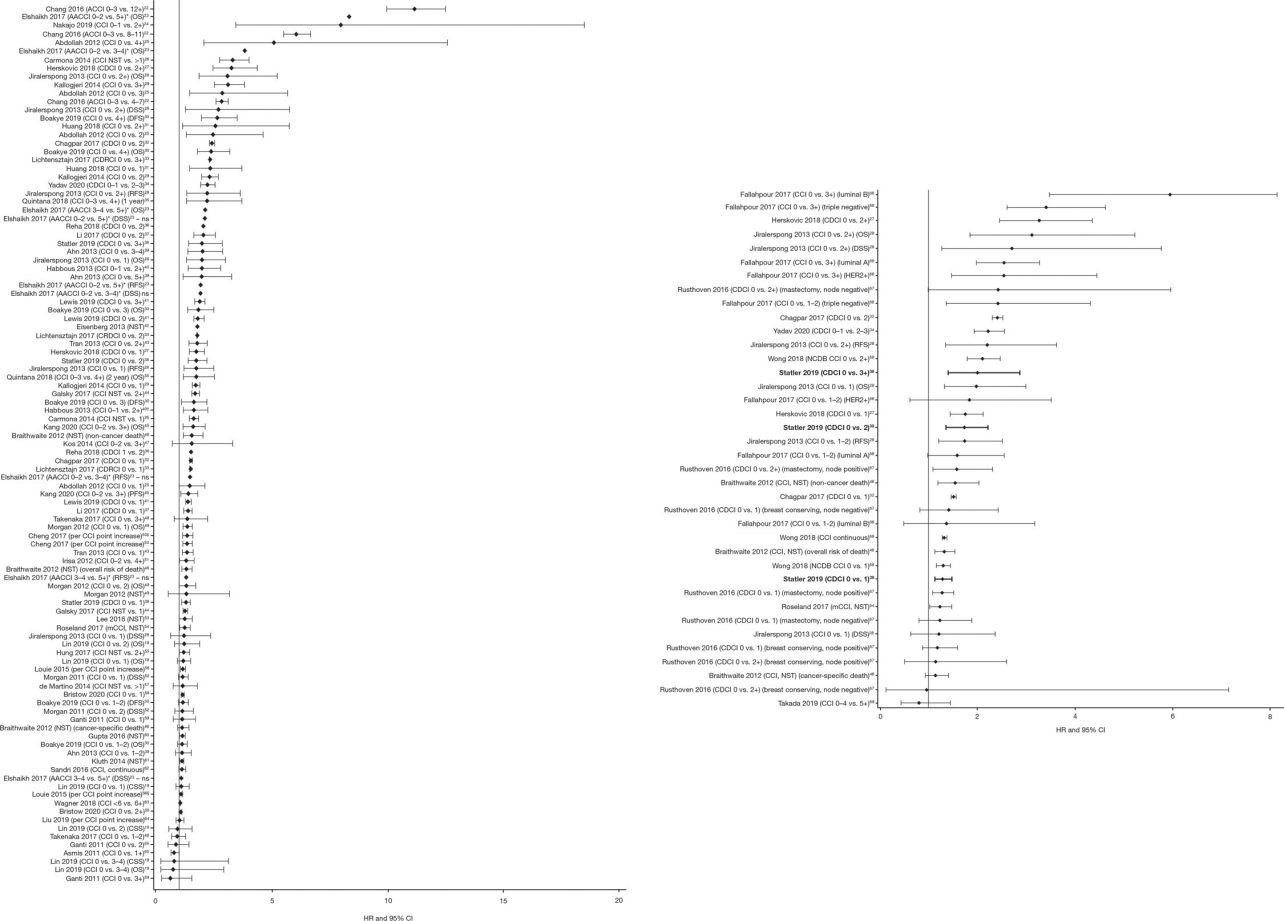
HRs for survival outcomes by CCI score for a) any cancer, b) any breast cancer. HRs for any survival outcome are included in the forest plot. Only one article reported the use of a comorbidity index in HER2+ breast cancer–this article is indicated in the above plot by use of boldface text in the lead author’s surname and analysis detail (Statler 2019). Note: These figures only include the following: (1) multivariate HRs, when both univariate and multivariate were provided, (2) adjusted HRs, when unadjusted and adjusted were provided. Figures exclude the following: (1) HRs, where only univariate HRs were reported but multivariate HRs were reported to be “non-significant” without additional details, (2) estimates of risk that were not reported in HRs, (3) odds ratios, (4) HRs, where only subgroup analysis was reported and overall analysis was not (exception: breast cancer CCI plot where breast-cancer subgroups were allowed). Unless specified as “ns” on the y-axis, HRs without 95% CIs were significant (p < 0.05). *HRs reported as inverse in original article and inverted for inclusion in this figure; significance is not reported here. ^†^Patient-reported scores and standard scores, respectively. ^‡^Based on pre- and postoperative scores, respectively. ^§^Based on training cohort and validation cohort, respectively. *ACCI*, *AACCI* age-adjusted Charlson Comorbidity Index, *CCI* Charlson Comorbidity Index, *CDCI* Charlson-Deyo Comorbidity Index, *CDRCI* Deyo and Romano adapted CCI, *CI* confidence interval, *DFS*, disease-free survival, *DSS*, disease-specific survival, *HER2+* human epidermal growth factor receptor-2-positive, *HR* hazard ratio, *mCCI* modified CCI, *ns* non-significant, *NST* not stated, *OS* overall survival, *RFS* relapse-free survival.

Predictive validity was the most common type of validity testing of comorbidity indices reported in the articles, with 179 (71.0%) “any cancer” studies presenting an outcome prediction. The most common outcome prediction was survival/mortality (145/179 [81.0%] articles). Of these articles, 80 reported HRs for survival outcomes and these are presented in forest plots. Fig 2A and 2B show forest plots of HRs and 95% confidence intervals (CIs) for all survival outcomes for CCI and CCI derivative indices for: a) any cancer (45 articles [19, 22–65]), and b) breast cancer including HER2+ breast cancer (11 articles [27, 28, 32, 34, 38, 46, 54, 66–69]). HRs are based on survival or mortality for a lower CCI score (reference) versus a higher score (see axis labeling in Fig 2A and 2B). Most of these studies stated that a HR > 1 indicated worse outcomes with increasing comorbidity scores, whether the outcome was reported in terms of survival or mortality. In a few studies, the direction of this relationship was not clearly stated. Forest plots of HRs and 95% CIs for survival outcomes for the two other most commonly used comorbidity indices (HCT-CI, ACE-27), and all other comorbidity indices combined, are presented in S1 Fig.

Table 4 shows the detailed predictive validity results for comorbidity indices based on survival outcomes for breast cancer and HER2+ breast cancer. Only one article was identified on HER2+ breast cancer [38]. Using the Charlson-Deyo Comorbidity Index (CDCI), a score of ≥ 1 was associated with significantly lower overall survival (OS) compared with a score of zero. In a subgroup of patients treated with chemotherapy/hormonal therapy and HER2+-targeted therapy, the effect of comorbidity burden on OS was not significant for a score of 1 or 2 versus zero, and only reached statistical significance for a score of ≥ 3 versus zero (Table 4) [38].

**Table 4:** Survival and mortality predictive validity results for breast cancer and HER2+ breast cancer by comorbidity index and outcome.

Citation	*N*	Comorbidity index	Outcome predicted	Outcome results
Clough-Gorr 2010 **[85]**	660	CCI	Mortality	In the total study population, CCI was significantly associated with poor treatment tolerance:
				• CCI 1+ (crude OR 2.75, 95% CI 1.38–5.49)
				• CCI 1+ (adjusted OR 2.49, 95% CI 1.18–5.25)
				In the total study population, CCI was significantly associated with mortality:
				• CCI 1+ (crude HR 1.73, 95% CI 1.30–2.31)
				• CCI 1+ (adjusted HR 1.38, 95% CI 1.01–1.88)
Wong 2018 **[68]**	11,243 (breast cancer)	CDCI	5-year survival	In the subpopulation of patients with breast cancer, NCDB CCI scores were significantly associated with postoperative 5-year survival:
				• CCI 1 vs. 0 (adjHR 1.30, 95% CI 1.17–1.44)
				• CCI 2+ vs. 0 (adjHR 2.10, 95% CI 1.79–2.46)
Takada 2019 **[69]**	75	CCI	PFS	In univariate analysis, Charlson score was not significantly associated with PFS:
				• CCI 4+ vs. < 4 (HR 0.800, 95% CI 0.434–1.450, p = 0.462)
Fallahpour 2017 **[66]**	29,833	CCI	BCSS	Charlson score 1–2 (vs. 0) was only significantly associated with BCSS in triple-negative cancer:
				• Luminal A (HR 1.59, 95% CI 0.98–2.54)
				• Luminal B (HR 1.37, 95% CI 0.49–3.17)
				• HER2+ (HR 1.84, 95% CI 0.62–3.51)
				• Triple negative (HR 2.42, 95% CI 1.36–4.31)
				Charlson score 3+ (vs. 0) was significantly associated with BCSS in all molecular subtypes:
				• Luminal A (HR 2.54, 95% CI 1.98–3.27)
				• Luminal B (HR 5.94, 95% CI 3.48–8.13)
				• HER2+ (HR 2.54, 95% CI 1.47–4.44)
				• Triple negative (HR 3.41, 95% CI 2.61–4.62)
Herskovic 2018 **[27]**	61,395	CDCI	OS	In univariate analysis, lower Charlson-Deyo scores were significantly associated with improved OS:
				• Score 1 vs. 0 (HR 1.88, 95% CI 1.75–2.03, p < 0.001)
				• Score 2+ vs. 0 (HR 3.51, 95% CI 3.10–3.97, p < 0.001)
				In multivariate analysis, lower Charlson-Deyo scores were significantly associated with improved OS:
				• Score 1 vs. 0 (HR 1.75, 95% CI 1.44–2.11, p < 0.001)
				• Score 2+ vs. 0 (HR 3.27, 95% CI 2.45–4.36, p < 0.001)
Jiralerspong 2013 **[28]**	6,342	CCI	OS, RFS, BCSS	Higher Charlson score (1 vs. 0) was significantly associated with:
				• RFS (HR 1.74, 95% CI 1.21–2.51, p = 0.003)
				• OS (HR 1.98, 95% CI 1.32–2.99, p = 0.001)
				• BCSS (HR 1.22, 95% CI 0.63–2.36, p = 0.562)
				Lower Charlson score (2+ vs. 0) was significantly associated with:
				• RFS (HR 2.20, 95% CI 1.34–3.62, p = 0.002)
				• OS (HR 3.11, 95% CI 1.85–5.22, p < 0.001)
				• BCSS (HR 2.70, 95% CI 1.27–5.76, p = 0.010)
Statler 2019 **[38]**	6,234	CDCI	OS	Charlson-Deyo scores were significantly associated with OS:
				• Score 1 vs. 0 (HR 1.29, 95% CI 1.13–1.47, p < 0.001)
				• Score 2 vs. 0 (HR 1.74, 95% CI 1.36–2.22, p < 0.001)
				• Score 3+ vs. 0 (HR 2.00, 95% CI 1.40–2.87, p < 0.001)
				Charlson-Deyo scores were non-significantly associated with OS (except score 3+) in patients treated with chemotherapy and HER2-targeted therapy
				• Score 1 vs. 0 (HR 1.16, 95% CI 0.88–1.54, p = 0.28)
				• Score 2 vs. 0 (HR 1.19, 95% CI 0.65–2.19, p = 0.58)
				• Score 3+ vs. 0 (HR 4.14, 95% CI 1.93–8.89, p < 0.001)
Yadav 2020 **[34]**	10,873	CCI	OS	• In univariate analysis, Charlson score (2–3 vs. 0–1) was significantly associated with OS (HR 3.26, 95% CI 2.84–3.74, p < 0.001)
				• In multivariate analysis, Charlson score (2–3 vs. 0–1) was significantly associated with OS (HR 2.22, 95% CI 1.93–2.55, p < 0.001)
Braithwaite 2012 **[46]**	2,272	CCI	BCSS, nBCSS, ACS	In multivariate analysis, Charlson score > 0 was significantly associated with increased risk of:
				• ACS (HR 1.32, 95% CI 1.13–1.54)
				• nBCSS (HR 1.55, 95% CI 1.19–2.02)
				• *Not* BCSS (HR 1.14, 95% CI 0.93–1.41)
				CCI score and outcomes by age: CCI had increased effect on outcomes in younger patients compared to older patients:
				• OS (HR 1.49, 95% CI 0.91–2.43 for age < 50; HR 1.42, 95% CI 1.12–1.80 for ages 50–64; and HR 1.17, 95% CI 0.94–1.47 for ages 65–79)
				• Non-breast-cancer survival (HR 1.84, 95% CI 1.11–3.05, for ages 50–64, and HR 1.29, 95% CI 0.94–1.78 for ages 65–79)
				In multivariate analysis, the association between CCI and non-breast-cancer mortality was higher among younger women:
				• 50–64 years (HR 1.84, 95% CI 1.11–3.05) vs. ≥ 65 (adjusted HR 1.29, 95% CI 0.94–1.78)
				In adjusted models, the effect of CCI on OS varied by stage of disease:
				• Stage I (HR 1.65, 95% CI 1.26–2.16)
				• Stage IIa (HR 1.33, 95% CI 1.02–1.74)
				• Stage IIb (HR 1.09, 95% CI 0.80–1.49)
				• Stage III (HR 0.53, 95% CI 0.23–1.25)
Roseland 2017 **[54]**	542	mCCI	10-year survival	Charlson score significantly associated with 10-year survival in patients with ER/PR- breast cancer:
				• HR 1.23, 95% CI 1.03–1.47, p = 0.02
Chagpar 2017 **[32]**	157,584	CDCI	OS	Charlson score significantly associated with OS:
				• CDCI 1 vs. 0 (HR 1.510, 95% CI 1.465–1.556)
				• CDCI 2 vs. 0 (HR 2.408, 95% CI 2.303–2.518)

*ACS* all-cause survival, *BCSS* breast cancer-specific survival, *CCI* Charlson Comorbidity Index, *CDCI* Charlson-Deyo Comorbidity Index, *CI* confidence interval, *ER* estrogen receptor, *HER2+* human epidermal growth factor receptor-2-positive, *HR* hazard ratio, *mCCI* modified CCI, *nBCSS* non-breast cancer-specific survival, *NCDB* National Cancer Database, *OR* odds ratio, *OS* overall survival, *PFS* progression-free survival, *PR* progesterone receptor, *RFS* relapse-free survival.

Other outcomes were predicted using comorbidity indices; however, due to the extent of these results and the limited capacity to incorporate all of them into this review, they are not reported here. A description of these outcomes can be found in S2 Table.

## 4. Discussion

The main objective of our review was to identify validated comorbidity indices that have been studied in patients with any type of cancer, breast cancer, and HER2+ breast cancer. Following a systematic review of published literature, we found the most commonly used validated comorbidity index in these patient groups was the CCI (or derivatives). The most common cancers studied in the articles were hematologic and breast cancers. This result is not surprising given that in 2019, breast cancer was estimated to be the most commonly diagnosed cancer type in the US and so is clearly an important focus of research and publications (including research involving comorbidity index validation). Furthermore, because we conducted two literature searches specifically looking at breast cancer in addition to the search for “any cancer”, it is not surprising that the searches returned more breast-cancer references. In addition, we also grouped all hematologic cancers together, which collectively also accounts for a large proportion of all cancers in the US [70, 71]. The CCI is a general comorbidity measure developed in 1987 by Charlson and colleagues [5]. It was initially developed using hospital notes data from a cohort of 604 patients and was then validated using a cohort of patients with breast cancer between 1962 and 1969 [5]. Subsequent developments have enabled the use of administrative data and patient self-reporting, and have seen the CCI used in many different disease states [72–75]. Furthermore, the CCI has been used as the basis for many other general comorbidity indices, as well as disease-specific comorbidity indices, including the HCT-CI [76].

After the CCI or CCI derivatives, the next most commonly used comorbidity indices in the identified studies were the HCT-CI and ACE-27. In our review, the HCT-CI was used to evaluate comorbidities in 72.9% of all articles evaluating hematologic cancers. The HCT-CI has become a widely used validated tool to predict outcomes in patients with hematologic cancers following transplant [77]. The ACE-27 was another commonly used comorbidity index in our data set. ACE-27, derived from the Kaplan-Feinstein Comorbidity Index, is a validated chart-based comorbidity index specifically designed for patients with cancer [6].

Within the data set, predictive validity was by far the most commonly reported type of validity and the CCI had the largest amount of evidence for predictive validity over the past 10 years. The majority of articles used survival outcomes to evaluate predictive validity. The forest plots of HRs for survival outcomes clearly show that increasing comorbidity burden (represented by a higher score on the comorbidity index) is generally associated with a greater risk of mortality across cancer types and comorbidity indices, with some exceptions (Figs 2A and 2B and S1). The consistency in these results highlights the negative impact of comorbidities in patients with cancer.

Only the CCI and derivatives were validated in patients with breast cancer. This suggests that a CCI-based index should be strongly considered when measuring comorbidities in breast-cancer populations, or other comorbidity indices should undergo validation before being used in these populations. Our review identified only one article that validated a comorbidity index (the CDCI) in the subgroup of patients with HER2+ breast cancer [38]. This lack of evidence makes it impossible to make a strong recommendation on the use of a particular comorbidity index in this population, and indicates a need for future research to validate comorbidity indices in this population.

Current breast-cancer treatment guidelines have limited recommendations based on comorbidities and comorbidity scores [78, 79]. The guidelines for adjuvant systemic therapy in early-stage operative breast cancer do recommend considering comorbidities in decision-making, but there is no guidance on choice of comorbidity index or how to tailor treatment based on comorbidities [80, 81]. Interestingly, the 2014 ASCO treatment guidelines in HER2- breast cancer stated that creating evidence-based recommendations on treatments is challenging in patients with comorbidities, especially as evidence for drug efficacy is often from clinical trials, which generally exclude patients with comorbidities. These guidelines noted that future treatment guidelines should provide information on how to apply recommendations for patients with comorbidities [82]. However, there is still a lack of guidance on how to account for comorbidities in the management of patients with breast cancer.

In addition to the effect of comorbidities on survival outcomes, we found comorbidity scores were used to assess a range of other outcomes in any cancer, such as adverse events and treatment tolerance, although the individual outcomes were reported by very few studies (S2 Table) making it impossible to evaluate effectively in our review. Other studies have found that patients with cancer with a higher comorbidity burden may experience delayed diagnosis, suboptimal treatment, and increased postoperative complications [1, 83]. For example, patients with HER2+ breast cancer and comorbidities may not complete treatment [2] and/or may experience significantly altered physician treatment decisions regarding HER2+-targeted treatment. While there is a need to further investigate the relationship between comorbidities, treatment choice, and outcomes, it is already evident that comorbidities have a large impact on cancer (specifically breast cancer) management. A “call to action” published by Sarfati et al. in 2016 focused on several strategies to address comorbidities, including improving the evidence base for cancer treatment decisions in patients with comorbidities, improving the measurement of comorbidities in patients with cancer, and developing better tools for clinicians [84].

This review has several limitations. The dates of the literature searches were January 1, 2010 to February 5, 2020. As the CCI was developed in 1987 and derivative versions not long after, pivotal studies on these comorbidity indices, especially those assessing their validity, prior to 2010 were missed in our literature search. Nevertheless, by using a later timeframe, we will have captured the most recent developments in comorbidity indices and in more contemporary studies of patients with cancer, which is important as treatments have impacted survival across cancers. A further potential limitation is publication bias, as studies that found a significant association between comorbidity index scores and outcomes may have been more likely to be published than those that found no association. However, the primary focus of many studies was not the predictive ability of the comorbidity index so the decision to publish is unlikely to have been based on this outcome. An additional limitation of this study was the inability to evaluate and compare different methods used to collect comorbidity information. Various articles used chart review, patient interviews, or ICD-9/ICD-10 code to identify comorbidities, but a large proportion of articles included in this review did not explicitly report their method of collecting the comorbidity information.

It was not possible to run a meta-analysis to provide a weighted average of the HRs presented in Figs 1 and 2 because of the heterogeneity of the studies. The heterogeneity of studies relates to the study populations and inclusion criteria (in both the “any cancer” and breast cancer groups of articles), particularly with respect to patient age ranges included, years of inclusion, molecular subtypes of disease included (including HR and HER2 status), stages of disease included, and sex/gender of patients included. Furthermore, the articles varied greatly on their categorization of comorbidity scores. For example, some articles categorized patients into CCI scores of 0, 1, or 2+ while others categorized patients into 0–1 or 2+, and still others categorized patients into 0–5 or 6+. In total, there were too many categorizations of comorbidity scores to perform meaningful meta-analysis.

Finally, it is worth noting that comorbidity indices such as the CCI may have significant limitations to their usefulness, especially in cancer populations. This may be due to underlying interactions between comorbidities, survival outcomes, and certain medications (whether used to treat the cancer or a comorbidity). For example, Kichenadasse et al. [86] examined the relationship between obesity, cancer treatment, and survival in patients diagnosed with non-small cell lung cancer enrolled in clinical trials and found that in patients treated with atezolizumab, those with obesity (BMI≥30) showed better overall survival compared to those with lower BMIs. Furthermore, Tang et al. [87] performed a literature review and meta-analysis to evaluate the association between metformin use and breast cancer mortality, and found that metformin use in breast cancer patients with diabetes was associated with significantly reduced all-cause mortality. Findings such as these indicate that the relationship between comorbidities and outcomes in patients with cancer may be more complicated than can be captured with comorbidity indices.

In conclusion, CCI-based indices are the most commonly used and validated comorbidity indices in any cancer, and the only indices used and validated in published breast-cancer research. Validation of comorbidity indices is mainly based on prediction of survival outcomes, which confirms the association between comorbidity burden and decreased survival. Due to insufficient evidence of validated comorbidity indices in patients with HER2+ breast cancer, further research on comorbidity indices in HER2+ breast cancer is warranted.

## Supporting information

S1 Fig Forest plots of HRs and 95% CIs for all survivor outcomes combined by comorbidity score for: a) HCT-CI [1–17], b) ACE-27 [18–25], or c) other comorbidity index for any cancer [26–34]. Note: These figures only include the following: (1) multivariate HRs, when both univariate and multivariate were provided, (2) adjusted HRs, when unadjusted and adjusted were provided. Figures exclude the following: (1) HRs, where only univariate HRs were reported but multivariate HRs were reported to be “non-significant” without additional details, (2) estimates of risk that were not reported in HRs, (3) odds ratios, (4) HRs, where only subgroup analysis was reported and overall analysis was not. Unless specified as “ns” on the y-axis, HRs without 95% CIs were significant (p < 0.05). *ACE-27* Adult Comorbidity Evaluation-27, *ACM* all-cause mortality, *aPCCI*, age-adjusted Prostate Cancer Comorbidity Index, *CI* confidence interval, *CIRS* Cumulative Illness Rating Scale, *CIRS-G* Cumulative Illness Rating Scale-Geriatric, *CRCCI* Colorectal Cancer Comorbidity Index, *ECIS* Elixhauser Comorbidity Index Score, *FCI* Freiburg Comorbidity Index, *HCT-CI* Hematopoietic Cell Transplant Comorbidity Index, *HN-CCI* Head and Neck Comorbidity Index, *HR* hazard ratio, *NRM* non-relapse mortality, *ns* non-significant, *NST* not stated, *OHNCI* Osaka Head and Neck Comorbidity Index, *OS* overall survival, *PCCI* Prostate Cancer Comorbidity Index, *PFS* progression-free survival, *WUHNCI* Washington University Head and Neck Comorbidity Index. (a) *HRs reported as inverse in original article and inverted for inclusion in this figure. (b) *No p-value or CIs reported. (c) *Two measures, based on validation cohort and initial cohort.
(TIF)

S1 Table (a) Search strategy using BIOSIS, Embase, and MEDLINE literature databases (any cancer). Databases: BIOSIS previews: 1993 to 2020 Week 11; Embase: 1974 to February 5, 2020; Ovid MEDLINE all: 1946 to February 5, 2020. (b) Search strategy using PubMed database (any cancer). (c) Search strategy using BIOSIS, Embase, and MEDLINE literature databases (breast cancer). Databases: BIOSIS previews: 1993 to 2020 Week 11; Embase: 1974 to February 5, 2020; Ovid MEDLINE all: 1946 to February 5, 2020. (d) Search strategy using PubMed database (breast cancer and HER2+ breast cancer). (e) Search strategy using BIOSIS, Embase, and MEDLINE literature databases (HER2+ breast cancer). Databases: BIOSIS previews: 1993 to 2020 Week 11; Embase: 1974 to February 5, 2020; Ovid MEDLINE all: 1946 to February 5, 2020.
(ZIP)

S2 Table Additional outcomes predicted using comorbidity indices. (DOCX)

S3 Table Risk of bias evaluation for included articles. (DOCX)

S1 Checklist PRISMA 2009 checklist. (DOC)
