# Supplementary figures and images for: Use of comorbidity indices in patients with any cancer, breast cancer, and human epidermal growth factor receptor-2-positive breast cancer: A systematic review
Source: PLoS One. 2021 Jun 18;16(6):e0252925. doi: 10.1371/journal.pone.0252925 (PMC8213062; doi:10.1371/journal.pone.0252925)

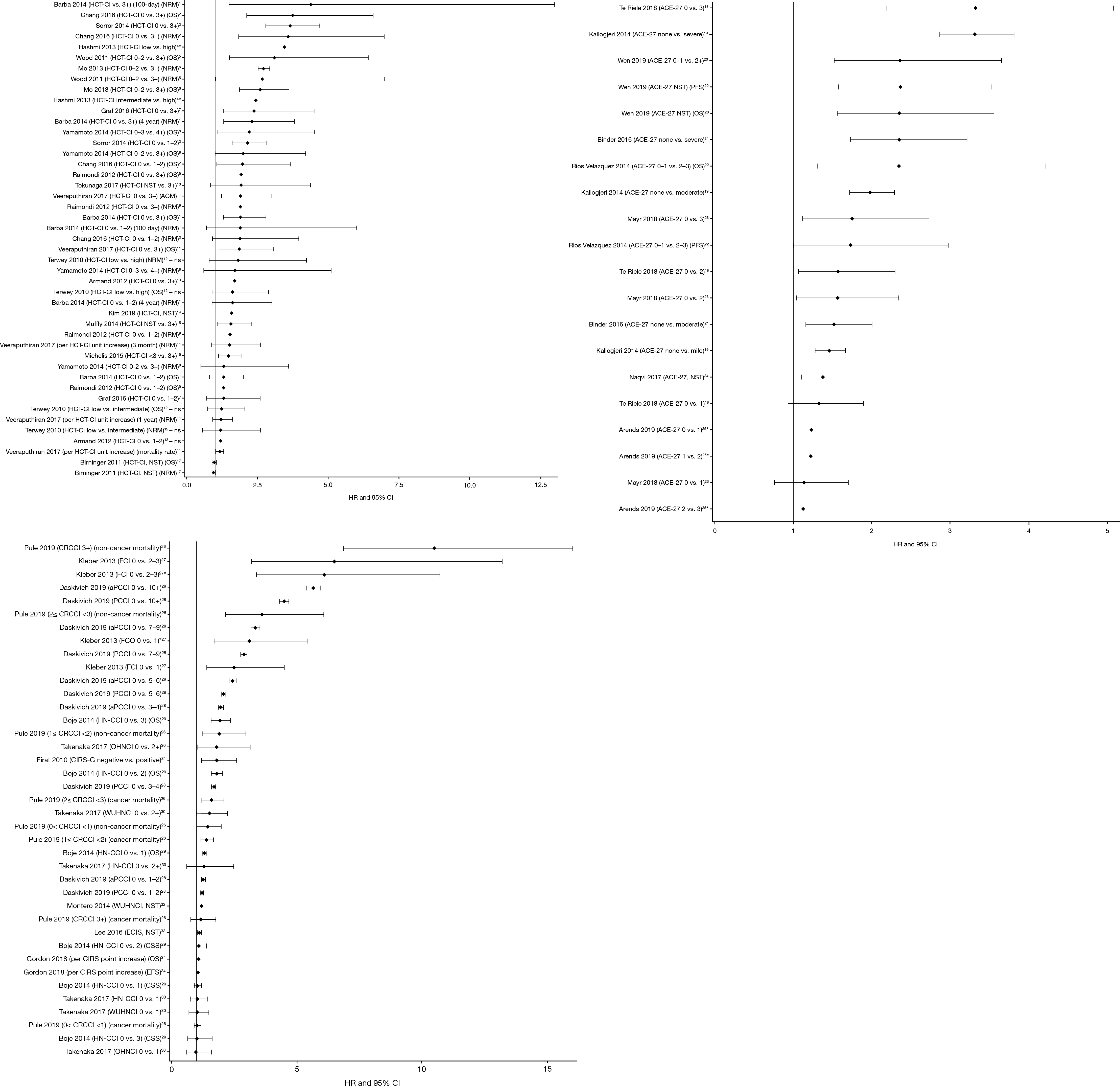

Supplement: S1 Fig — Forest plots of HRs and 95% CIs for all survivor outcomes combined by comorbidity score for: a) HCT-CI [1–17], b) ACE-27 [18–25], or c) other comorbidity index for any cancer [26–34]. Note: These figures only include the following: (1) multivariate HRs, when both univariate and multivariate were provided, (2) adjusted HRs, when unadjusted and adjusted were provided. Figures exclude the following: (1) HRs, where only univariate HRs were reported but multivariate HRs were reported to be “non-significant” without additional details, (2) estimates of risk that were not reported in HRs, (3) odds ratios, (4) HRs, where only subgroup analysis was reported and overall analysis was not. Unless specified as “ns” on the y-axis, HRs without 95% CIs were significant (p < 0.05). ACE-27 Adult Comorbidity Evaluation-27, ACM all-cause mortality, aPCCI, age-adjusted Prostate Cancer Comorbidity Index, CI confidence interval, CIRS Cumulative Illness Rating Scale, CIRS-G Cumulative Illness Rating Scale-Geriatric, CRCCI Colorectal Cancer Comorbidity Index, ECIS Elixhauser Comorbidity Index Score, FCI Freiburg Comorbidity Index, HCT-CI Hematopoietic Cell Transplant Comorbidity Index, HN-CCI Head and Neck Comorbidity Index, HR hazard ratio, NRM non-relapse mortality, ns non-significant, NST not stated, OHNCI Osaka Head and Neck Comorbidity Index, OS overall survival, PCCI Prostate Cancer Comorbidity Index, PFS progression-free survival, WUHNCI Washington University Head and Neck Comorbidity Index. (a) *HRs reported as inverse in original article and inverted for inclusion in this figure. (b) *No p-value or CIs reported. (c) *Two measures, based on validation cohort and initial cohort. (TIF) [file pone.0252925.s001.tif]
