# Supplementary material for: Use of comorbidity indices in patients with any cancer, breast cancer, and human epidermal growth factor receptor-2-positive breast cancer: A systematic review
Source: PLoS One. 2021 Jun 18;16(6):e0252925. doi: 10.1371/journal.pone.0252925 (PMC8213062; doi:10.1371/journal.pone.0252925)
Supplement: S1 Table — (a) Search strategy using BIOSIS, Embase, and MEDLINE literature databases (any cancer). Databases: BIOSIS previews: 1993 to 2020 Week 11; Embase: 1974 to February 5, 2020; Ovid MEDLINE all: 1946 to February 5, 2020. (b) Search strategy using PubMed database (any cancer). (c) Search strategy using BIOSIS, Embase, and MEDLINE literature databases (breast cancer). Databases: BIOSIS previews: 1993 to 2020 Week 11; Embase: 1974 to February 5, 2020; Ovid MEDLINE all: 1946 to February 5, 2020. (d) Search strategy using PubMed database (breast cancer and HER2+ breast cancer). (e) Search strategy using BIOSIS, Embase, and MEDLINE literature databases (HER2+ breast cancer). Databases: BIOSIS previews: 1993 to 2020 Week 11; Embase: 1974 to February 5, 2020; Ovid MEDLINE all: 1946 to February 5, 2020. (ZIP) [file pone.0252925.s002.zip › S1a_Table.docx]

**S1a Table. Search strategy using BIOSIS, Embase, and MEDLINE literature databases (any cancer).**

| # | Searches | Results |
| --- | --- | --- |
| 1 | (comorbidity adj2 (index or indexes or indices)).ti,ab. | 28,990 |
| 2 | ((comorbidity or comortality) adj2 (scor??? or scal??? or assess????? or measur?????? or tool? or analys?s)).ti,ab. | 16,794 |
| 3 | exp comorbidity assessment/ or *comorbidity/ | 38,104 |
| 4 | 1 or 2 or 3 | 64,264 |
| 5 | validation study/ or validation process/ | 216,165 |
| 6 | ((valid or validated or validation or evaluate? or reliable or reliability) adj2 (profil?? or instrument? or scale??? or scor??? or measur?????? or questionnaire? or assess????? or index or indexes or indices or tool? or analys?s)).ti,ab. | 520,292 |
| 7 | Validation Studies as Topic/ | 83,880 |
| 8 | 5 or 6 or 7 | 714,492 |
| 9 | 4 and 8 | 2,290 |
| 10 | cohort analysis/ or meta analysis/ or case control study/ or controlled study/ or observational study/ or case-control studies/ or clinical study/ or clinical trial/ | 9,728,879 |
| 11 | ((non-interventional or non-interventional or cohort or meta or case control or clinical or controlled or observational) adj2 (stud??? or trial? or analys?s)).mp. | 14,222,960 |
| 12 | 10 or 11 | 14,222,960 |
| 13 | neoplasm/ or malignant neoplasm/ or carcinoma/ or sarcoma/ or melanoma/ or lymphoma/ or leukemia/ or neoplasms/ or metastasis/ | 1,752,341 |
| 14 | (carcinoma or sarcoma or melanoma or lymphoma or leukemia or neoplasm or cancer or malignancy or malignant or metastasis).mp. | 10,157,089 |
| 15 | breast cancer/ or lung cancer/ or prostate cancer/ or colorectal cancer/ or melanoma/ or bladder cancer/ or kidney cancer/ or endometrial cancer/ or pancreatic cancer/ or thyroid cancer/ or liver cancer/ | 2,099,334 |
| 16 | ((breast or lung or prostate or colorectal or bladder or kidney or endometrial or pancreatic or thyroid or liver) adj2 cancer?).mp. | 2,896,295 |
| 17 | melanoma.mp. | 437,831 |
| 18 | (("Stage 1" or "Stage I" or "Stage i" or "Stage 2" or "Stage II" or "Stage ii" or "Stage 3" or "Stage III" or "Stage iii" or "Stage 4" or "Stage IV") adj2 cancer?).ti,ab. | 32,991 |
| 19 | (locally advanced adj2 cancer?).mp. | 38,281 |
| 20 | cancer staging/ or neoplasm staging/ | 484,395 |
| 21 | 13 or 14 or 15 or 16 or 17 or 18 or 19 or 20 | 10,428,294 |
| 22 | (9 and 12 and 21) or (9 and 21) | 748 |
| 23 | limit 22 to human | 705 |
| 24 | 22 and human medicine/ | 96 |
| 25 | 23 or 24 | 709 |
| 26 | 25 not ((preclinical or nonhuman or animal) adj2 (experiment? or model? or stud???)).kw,sh. | 709 |
| 27 | 26 not (case report* or case stud* or case series).kw,sh. | 706 |
| 28 | 27 not (editorial or letter or conference abstract or conference paper).pt. | 441 |
| 29 | 28 not (abstract or poster or commentary).kw,sh. | 439 |
| 30 | limit 29 to yr=2010-2020 | 333 |
| 31 | remove duplicates from 30 | 235 |
| 32 | remove a further single duplicate reference (remained after exportation and was eliminated from final results) | 234 |

Databases: BIOSIS previews: 1993 to 2020 Week 11; Embase: 1974 to February 5, 2020; Ovid MEDLINE all: 1946 to February 5, 2020.
