# Supplementary material for: Use of comorbidity indices in patients with any cancer, breast cancer, and human epidermal growth factor receptor-2-positive breast cancer: A systematic review
Source: PLoS One. 2021 Jun 18;16(6):e0252925. doi: 10.1371/journal.pone.0252925 (PMC8213062; doi:10.1371/journal.pone.0252925)
Supplement: S1 Table — (a) Search strategy using BIOSIS, Embase, and MEDLINE literature databases (any cancer). Databases: BIOSIS previews: 1993 to 2020 Week 11; Embase: 1974 to February 5, 2020; Ovid MEDLINE all: 1946 to February 5, 2020. (b) Search strategy using PubMed database (any cancer). (c) Search strategy using BIOSIS, Embase, and MEDLINE literature databases (breast cancer). Databases: BIOSIS previews: 1993 to 2020 Week 11; Embase: 1974 to February 5, 2020; Ovid MEDLINE all: 1946 to February 5, 2020. (d) Search strategy using PubMed database (breast cancer and HER2+ breast cancer). (e) Search strategy using BIOSIS, Embase, and MEDLINE literature databases (HER2+ breast cancer). Databases: BIOSIS previews: 1993 to 2020 Week 11; Embase: 1974 to February 5, 2020; Ovid MEDLINE all: 1946 to February 5, 2020. (ZIP) [file pone.0252925.s002.zip › S1b_Table.docx]

**S1b Table.** Search strategy using PubMed database (any cancer)

| **Literature search strategy – PubMed (any cancer)** |
| --- |
| ((((((((((("valid"[All Fields] OR "validate"[All Fields]) OR "validated"[All Fields]) OR "validates"[All Fields]) OR "validating"[All Fields]) OR "validation"[All Fields]) OR "validational"[All Fields]) OR "validations"[All Fields]) OR "validator"[All Fields]) OR "validators"[All Fields]) OR "validities"[All Fields]) OR "validity"[All Fields]) AND ((((((((((("valid"[All Fields] OR "validate"[All Fields]) OR "validated"[All Fields]) OR "validates"[All Fields]) OR "validating"[All Fields]) OR "validation"[All Fields]) OR "validational"[All Fields]) OR "validations"[All Fields]) OR "validator"[All Fields]) OR "validators"[All Fields]) OR "validities"[All Fields]) OR "validity"[All Fields]) AND (((("comorbid"[All Fields] OR "comorbidity"[MeSH Terms]) OR "comorbidity"[All Fields]) OR "comorbidities"[All Fields]) OR "comorbids"[All Fields]) AND (((((((((((("abstracting and indexing"[MeSH Terms] OR ("abstracting"[All Fields] AND "indexing"[All Fields])) OR "abstracting and indexing"[All Fields]) OR "index"[All Fields]) OR "indexed"[All Fields]) OR "indexes"[All Fields]) OR "indexing"[All Fields]) OR "indexation"[All Fields]) OR "indexations"[All Fields]) OR "indexe"[All Fields]) OR "indexer"[All Fields]) OR "indexers"[All Fields]) OR "indexs"[All Fields]) AND ((((((((("cancer s"[All Fields] OR "cancerated"[All Fields]) OR "canceration"[All Fields]) OR "cancerization"[All Fields]) OR "cancerized"[All Fields]) OR "cancerous"[All Fields]) OR "neoplasms"[MeSH Terms]) OR "neoplasms"[All Fields]) OR "cancer"[All Fields]) OR "cancers"[All Fields]) AND ((("neoplasms"[MeSH Terms] OR "neoplasms"[All Fields]) OR "oncology"[All Fields]) OR "oncology s"[All Fields]) AND (((("patient s"[All Fields] OR "patients"[MeSH Terms]) OR "patients"[All Fields]) OR "patient"[All Fields]) OR "patients s"[All Fields]) |
| **= 298 results (remove duplicates from exported files = 249)** |
