# Supplementary material for: Use of comorbidity indices in patients with any cancer, breast cancer, and human epidermal growth factor receptor-2-positive breast cancer: A systematic review
Source: PLoS One. 2021 Jun 18;16(6):e0252925. doi: 10.1371/journal.pone.0252925 (PMC8213062; doi:10.1371/journal.pone.0252925)
Supplement: S1 Table — (a) Search strategy using BIOSIS, Embase, and MEDLINE literature databases (any cancer). Databases: BIOSIS previews: 1993 to 2020 Week 11; Embase: 1974 to February 5, 2020; Ovid MEDLINE all: 1946 to February 5, 2020. (b) Search strategy using PubMed database (any cancer). (c) Search strategy using BIOSIS, Embase, and MEDLINE literature databases (breast cancer). Databases: BIOSIS previews: 1993 to 2020 Week 11; Embase: 1974 to February 5, 2020; Ovid MEDLINE all: 1946 to February 5, 2020. (d) Search strategy using PubMed database (breast cancer and HER2+ breast cancer). (e) Search strategy using BIOSIS, Embase, and MEDLINE literature databases (HER2+ breast cancer). Databases: BIOSIS previews: 1993 to 2020 Week 11; Embase: 1974 to February 5, 2020; Ovid MEDLINE all: 1946 to February 5, 2020. (ZIP) [file pone.0252925.s002.zip › S1c_Table.docx]

**S1c Table. Search strategy using BIOSIS, Embase, and MEDLINE literature databases (breast cancer).**

| # | Searches | Results |
| --- | --- | --- |
| 1 | (comorbidity adj2 (index or indexes or indices)).ti,ab. | 28,990 |
| 2 | ((comorbidity or comortality) adj2 (scor??? or scal??? or assess????? or measur?????? or tool? or analys?s)).ti,ab. | 16,794 |
| 3 | exp comorbidity assessment/ or comorbidity/ | 376,110 |
| 4 | 1 or 2 or 3 | 391,783 |
| 5 | validation study/ or validation process/ | 216,165 |
| 6 | ((valid or validated or validation or evaluate? or reliable or reliability) adj2 (profil??? or instrument? or scal??? or scor??? or measure?????? or questionnaire? or assess????? or index or indexes or indices or tool? or analys?s)).ti,ab. | 499,127 |
| 7 | Validation Studies as Topic/ | 83,880 |
| 8 | 5 or 6 or 7 | 693,476 |
| 9 | 4 and 8 | 8,569 |
| 10 | cohort analysis/ or meta analysis/ or case control study/ or controlled study/ or observational study/ or case-control studies/ or clinical study/ or clinical trial/ | 9,728,879 |
| 11 | ((non-interventional or non-interventional or cohort or meta or case control or clinical or controlled or observational) adj2 (stud??? or trial? or analys?s)).mp. | 14,222,960 |
| 12 | 10 or 11 | 14,222,960 |
| 13 | breast cancer/ or triple negative breast cancer/ or inflammatory breast cancer/ or metastatic breast cancer/ or breast metastasis/ | 662,932 |
| 14 | medullary carcinoma/ or colloid carcinoma/ or breast carcinoma/ or intraductal carcinoma/ | 378,941 |
| 15 | ((breast or medullary or colloid or tubular or mucinous or intraductal or invasive ductal or ductal) adj2 carcinoma).mp. | 248,837 |
| 16 | (locally advanced adj2 breast cancer?).mp. | 8,208 |
| 17 | (breast cancer? or breast neoplasm? or inflammatory breast cancer? or inflammatory breast neoplasm? or triple negative breast cancer? or triple negative breast neoplasm? or unilateral breast cancer? or unilaterial breast neoplasm?).ti,ab,kw,sh. | 1,170,536 |
| 18 | (("Stage 1" or "Stage I" or "Stage i" or "Stage 2" or "Stage II" or "Stage ii" or "Stage 3" or "Stage III" or "Stage iii" or "Stage 4" or "Stage IV") adj2 breast cancer?).ti,ab. | 9,547 |
| 19 | Paget nipple disease.mp. | 7,479 |
| 20 | ((HR+ or hormone receptor positive or (ER+ or estrogen receptor positive) or (PR+ or progesterone receptor positive)) adj2 (breast cancer? or breast carcinoma?)).ti,ab. | 30,987 |
| 21 | ((HR- or hormone receptor negative or (ER- or estrogen receptor negative) or (PR- or progesterone receptor negative)) adj2 (breast cancer? or breast carcinoma?)).ti,ab. | 23,673 |
| 22 | 13 or 14 or 15 or 16 or 17 or 18 or 19 or 20 or 21 | 1,293,508 |
| 23 | (9 and 12 and 22) or (9 and 22) | 173 |
| 24 | limit 23 to human | 166 |
| 25 | 23 and human medicine/ | 4 |
| 26 | 24 or 25 | 166 |
| 27 | 26 not ((preclinical or nonhuman or animal) adj2 (experiment? or model? or stud???)).kw,sh. | 166 |
| 28 | 27 not (case report* or case stud* or case series).kw,sh. | 164 |
| 29 | 28 not (editorial or letter or conference abstract or conference paper).pt. | 123 |
| 30 | 29 not (abstract or poster or commentary).kw,sh. | 123 |
| 31 | limit 30 to yr=2010-2020 | 94 |
| 32 | remove duplicates from 31 | 78 |

Databases: BIOSIS previews: 1993 to 2020 Week 11; Embase: 1974 to February 5, 2020; Ovid MEDLINE all: 1946 to February 5, 2020.
