# Supplementary material for: Use of comorbidity indices in patients with any cancer, breast cancer, and human epidermal growth factor receptor-2-positive breast cancer: A systematic review
Source: PLoS One. 2021 Jun 18;16(6):e0252925. doi: 10.1371/journal.pone.0252925 (PMC8213062; doi:10.1371/journal.pone.0252925)
Supplement: S1 Table — (a) Search strategy using BIOSIS, Embase, and MEDLINE literature databases (any cancer). Databases: BIOSIS previews: 1993 to 2020 Week 11; Embase: 1974 to February 5, 2020; Ovid MEDLINE all: 1946 to February 5, 2020. (b) Search strategy using PubMed database (any cancer). (c) Search strategy using BIOSIS, Embase, and MEDLINE literature databases (breast cancer). Databases: BIOSIS previews: 1993 to 2020 Week 11; Embase: 1974 to February 5, 2020; Ovid MEDLINE all: 1946 to February 5, 2020. (d) Search strategy using PubMed database (breast cancer and HER2+ breast cancer). (e) Search strategy using BIOSIS, Embase, and MEDLINE literature databases (HER2+ breast cancer). Databases: BIOSIS previews: 1993 to 2020 Week 11; Embase: 1974 to February 5, 2020; Ovid MEDLINE all: 1946 to February 5, 2020. (ZIP) [file pone.0252925.s002.zip › S1d_Table.docx]

**S1d Table.** Search strategy using PubMed database (breast cancer and HER2+ breast cancer)

| **Literature search strategy – PubMed (breast cancer/HER2+ breast cancer)** |
| --- |
| ("humans"[MeSH Terms] OR "humans"[All Fields] OR "human"[All Fields]) AND ("erbb receptors"[MeSH Terms] OR ("erbb"[All Fields] AND "receptors"[All Fields]) OR "erbb receptors"[All Fields] OR ("epidermal"[All Fields] AND "growth"[All Fields] AND "factor"[All Fields] AND "receptor"[All Fields]) OR "epidermal growth factor receptor"[All Fields]) AND positive[All Fields] AND ("breast neoplasms"[MeSH Terms] OR ("breast"[All Fields] AND "neoplasms"[All Fields]) OR "breast neoplasms"[All Fields] OR ("breast"[All Fields] AND "cancer"[All Fields]) OR "breast cancer"[All Fields]) AND ("comorbidity"[MeSH Terms] OR "comorbidity"[All Fields] OR "comorbidities"[All Fields]) |
| **= 66 results (-1 duplicate result in HER2+ breast cancer = 65; -2 duplicate results in breast cancer = 64)** |
