# Supplementary material for: Use of comorbidity indices in patients with any cancer, breast cancer, and human epidermal growth factor receptor-2-positive breast cancer: A systematic review
Source: PLoS One. 2021 Jun 18;16(6):e0252925. doi: 10.1371/journal.pone.0252925 (PMC8213062; doi:10.1371/journal.pone.0252925)
Supplement: S2 Table — (DOCX) [file pone.0252925.s003.docx]

**S2 Table.** Additional outcomes predicted using comorbidity indices

| Outcome |
| --- |
| Cognitive decline |
| Complications |
| Disability |
| Disease progression |
| Disease symptoms |
| Fatigue/lack of energy |
| Functional independence |
| Hospital readmission |
| Length of hospital stay |
| Long-term care stay |
| Medication use |
| Mental and physical capabilities |
| Morbidity |
| Postoperative delirium |
| Quality of life |
| Relapse |
| Remission |
| Sarcopenia/sarcopenic obesity |
| Sexual and erectile activity/function |
| Swallowing ability |
| Toxicity and adverse events |
| Treatment tolerance |
| Urinary incontinence |
