# Supplementary material for: Use of comorbidity indices in patients with any cancer, breast cancer, and human epidermal growth factor receptor-2-positive breast cancer: A systematic review
Source: PLoS One. 2021 Jun 18;16(6):e0252925. doi: 10.1371/journal.pone.0252925 (PMC8213062; doi:10.1371/journal.pone.0252925)
Supplement: S3 Table — (DOCX) [file pone.0252925.s004.docx]

# Risk of Bias Evaluation

| Newcastle Ottawa Criteria for Risk of Bias: Cohort Studies | | | | | | | | | | | | | | | | |
| --- | --- | --- | --- | --- | --- | --- | --- | --- | --- | --- | --- | --- | --- | --- | --- | --- |
| Citation | | **Representativeness of exposed cohort** | | **Selection of unexposed cohort** | **Ascertainment of exposure** | | **Demonstration that outcome of interest wasn’t present at study start** | | **Comparability of cohorts on basis of the design or analysis** | | **Assessment of outcome** | | **Was follow-up long enough for outcome to occur?** | **Adequacy of follow-up cohorts** | | **Total * (total possible = 9)** |
| Abdollah et al. Ann Surg Oncol. 2012 Jan;19(1):309-17. | | * | |  | * | | * | |  | | * | | * | * | | 6 |
| Aggerholm-Pedersen et al. Sarcoma. 2014;2014:690316. doi: 10.1155/2014/690316. Epub 2014 Feb 27. | | * | |  | * | | * | |  | | * | | * | * | | 6 |
| Ahiko et al. BMC Cancer. 19 (1) (no pagination), 2019. Article Number: 946. Date of Publication: 06 v 2019. | | * | |  | * | | * | |  | | * | | * | * | | 6 |
| Ahn et al. Clin Lung Cancer. 2013 v;14(6):644-50. | | * | |  | * | | * | |  | | * | | * |  | | 5 |
| Alexander et al. Br J Cancer. 2017 Aug 22;117(5):744-751. | | * | |  | * | | * | |  | | * | | * | * | | 6 |
| Alibhai et al. Journal of Geriatric Oncology. 8 (1) (pp 31-36), 2017. Date of Publication: 01 Jan 2017. | | * | |  | * | | * | |  | |  | | * | * | | 5 |
| Ambrogi et al. Eur Respir J. 2015 Apr;45(4):1089-97. | | * | | * | * | | * | | ** | | * | | * | * | | 9 |
| Arends et al. Laryngoscope. 2019 v 6:10.1002/lary.28345. doi: 10.1002/lary.28345. Online ahead of print. | | * | |  | * | | * | |  | |  | | * |  | | 4 |
| Armand et al. Blood. 2012 Jul 26;120(4):905-13. doi: 10.1182/blood-2012-03-418202. Epub 2012 Jun 18. | | * | | * | * | | * | | ** | |  | | * |  | | 7 |
| Arostegui et al. Clin Epidemiol. 2018 Mar 6;10:235-251. doi: 10.2147/CLEP.S146729. eCollection 2018. | | * | |  | * | | * | |  | | * | | * | * | | 6 |
| Aziz et al. Urologic Oncology: Seminars and Original Investigations. 32 (8) (pp 1141-1150), 2014. Date of Publication: 2014. | | * | |  | * | | * | |  | | * | | * |  | | 5 |
| Baitar et al. Journal of Geriatric Oncology 2015;6:401-410. | | * | |  | * | |  | |  | | * | | * | * | | 5 |
| Barba et al. Biol Blood Marrow Transplant. 2014 Jan;20(1):66-72. doi: 10.1016/j.bbmt.2013.10.011. Epub 2013 Oct 17. | | * | |  | * | | * | |  | | * | | * |  | | 5 |
| Barba et al. Biol Blood Marrow Transplant 23 2017 67-74 | | * | |  | * | | * | |  | |  | | * | * | | 5 |
| Barba et al. Biology of Blood and Marrow Transplantation. 16 (3) (pp 413-420), 2010. Date of Publication: March 2010. | | * | |  | * | | * | |  | |  | | * |  | | 4 |
| Beg et al. Am J Clin Oncol. 2018 Aug;41(8):766-771. doi: 10.1097/COC.0000000000000359. | | * | |  | * | | * | |  | | * | | * |  | | 5 |
| Bian et al. American Journal of Clinical Oncology: Cancer Clinical Trials. 39 (4) (pp 368-373), 2016. Date of Publication: 01 Aug 2016. | | * | |  | * | | * | |  | | * | |  |  | | 4 |
| Binder et al. American Journal of Obstetrics and Gynecology. 215 (6) (pp 766.e1-766.e9), 2016. Date of Publication: 01 Dec 2016. | | * | |  | * | | * | |  | | * | | * | * | | 6 |
| Birninger et al. Biol Blood Marrow Transplant. 2011 Dec;17(12):1822-32. doi: 10.1016/j.bbmt.2011.06.009. Epub 2011 Jun 25. | | * | |  | * | | * | |  | |  | | * |  | | 4 |
| Boakye et al. Cancers. 11 (10) ( pagination), 2019. Article Number: 1435. Date of Publication: October 2019. | | * | |  | * | | * | |  | | * | | * |  | | 5 |
| Boje et al. Radiother Oncol. 2014 Jan;110(1):91-7. doi: 10.1016/j.radonc.2013.11.009. Epub 2014 Jan 8. | | * | |  | * | |  | |  | | * | | * |  | | 4 |
| Braithwaite et al. Cancer Epidemiol Biomark Prev 21(7):1115–1125. doi:10. 1158/1055-9965.EPI-11-1228 | | * | |  | * | |  | |  | | * | | * | * | | 5 |
| Bratt et al. Eur Urol. 2015 Jul;68(1):53-8. doi: 10.1016/j.eururo.2014.12.026. Epub 2015 Mar 23. | | * | | * | * | | * | | ** | | * | | * |  | | 8 |
| Bristow et al. Obstet Gynecol. 2020 Feb;135(2):328-339. doi: 10.1097/AOG.0000000000003665. | | * | |  | * | | * | |  | | * | | * |  | | 5 |
| Buckstein et al. British Journal of Haematology. 174 (1) (pp 88-101), 2016. Date of Publication: 01 Jul 2016. | | * | |  | * | |  | |  | | * | | * | * | | 5 |
| Carmona et al. Int J Radiat Oncol Biol Phys. 2014 Jul 15;89(4):888-98. doi: 10.1016/j.ijrobp.2014.03.047. | | * | |  | * | | * | |  | | * | | * |  | | 5 |
| Casas Duran et al. Clin Transl Oncol. 2019 v 20:10.1007/s12094-019-02246-0. doi: 10.1007/s12094-019-02246-0. Online ahead of print. | | * | |  | * | | * | |  | | * | | * | * | | 6 |
| Chagpar et al. Ann Surg Oncol. 2017 Oct;24(10):3073-3081. doi: 10.1245/s10434-017-5936-x. Epub 2017 Aug 1. | | * | | * | * | | * | | ** | | * | | * |  | | 8 |
| Chang et al. PLoS ONE. 11 (2) ( pagination), 2016. Article Number: e0148076. Date of Publication: 01 Feb 2016. | | * | |  | * | | * | |  | | * | | * |  | | 5 |
| Chang et al. Breast. 43 (pp 48-54), 2019. Date of Publication: February 2019. | | * | |  | * | |  | | ** | | * | | * | * | | 7 |
| Chang et al. Leuk Lymphoma. 2016 Sep;57(9):2133-9. doi: 10.3109/10428194.2015.1124990. Epub 2016 Feb 9. | | * | |  | * | | * | |  | |  | | * |  | | 4 |
| Chavez-MacGregor et al. Journal of Clinical Oncology. 33 (19) (pp 2176-2183), 2015. Date of Publication: 01 Jul 2015. | | * | |  | * | |  | |  | | * | | * |  | | 4 |
| Chen et al. Journal of Surgical Oncology. 116 (4) (pp 533-544), 2017. Date of Publication: 15 Sep 2017. | | * | |  | * | | * | |  | | * | | * |  | | 5 |
| Cheng et al. Surgery (United States). 162 (6) (pp 1231-1240), 2017. Date of Publication: December 2017. | | * | |  | * | | * | |  | | * | | * | * | | 6 |
| Chiu et al. J Gastrointest Surg. 2018 Oct;22(10):1724-1731. doi: 10.1007/s11605-018-3833-7. Epub 2018 Jun 18. | | * | |  | * | |  | |  | |  | | * | * | | 4 |
| Chou et al. Journal of Cancer. 7 (1) (pp 14-21), 2016. Date of Publication: 2016. | | * | |  | * | | * | |  | | * | | * |  | | 5 |
| Chou et al. Cancer Med. 2015 v;4(11):1687-96. doi: 10.1002/cam4.518. Epub 2015 Aug 26. | | * | |  | * | | * | |  | | * | | * |  | | 5 |
| Cioffi et al. Tumori. 105 (2) (pp 168-173), 2019. Date of Publication: 01 Apr 2019. | | * | |  | * | | * | |  | |  | | * | * | | 5 |
| Clough-Gorr et al. Journal of Clinical Oncology. 28 (3) (pp 380-386), 2010. Date of Publication: 20 Jan 2010. | | * | |  | * | | * | |  | |  | | * | * | | 5 |
| Coffey et al. Bone Marrow Transplant. 2013 Sep;48(9):1253-6. doi: 10.1038/bmt.2013.31. Epub 2013 Mar 18. | |  | |  | * | |  | |  | | * | | * |  | | 3 |
| Collard et al. Journal of Surgical Research. 226 (pp 112-121), 2018. Date of Publication: June 2018. | |  | |  | * | | * | |  | | * | | * |  | | 4 |
| Cordas dos Santos et al. Biology of Blood and Marrow Transplantation. ( pagination), 2019. Date of Publication: 2019. | | * | | * | * | | * | | ** | |  | | * | * | | 8 |
| Cozzi et al. Andrologia. 2019 v;51(10):e13385. doi: 10.1111/and.13385. Epub 2019 Aug 18. | | * | |  |  | | * | |  | |  | | * |  | | 3 |
| Daniels et al. BMC Cancer. 2019 Sep 11;19(1):909. doi: 10.1186/s12885-019-6126-y. | | * | |  | * | | * | |  | |  | | * | * | | 5 |
| Dapkeviciute et al. Clinical Respiratory Journal. 14 (1) (pp 3-8), 2020. Date of Publication: 01 Jan 2020. | | * | |  | * | | * | |  | | * | | * |  | | 5 |
| Daskivich et al. The Journal of urology. 202 (3) (pp 518-524), 2019. Date of Publication: 01 Sep 2019. | | * | |  | * | | * | |  | | * | | * |  | | 5 |
| Datema et al. Head Neck. 2013 Sep;35(9):1232-7. doi: 10.1002/hed.23117. Epub 2012 Jul 30. | | * | |  | * | | * | | ** | | * | | * | * | | 8 |
| Davidoff et al. Medical Care. 52 (6) (pp 500-510), 2014. Date of Publication: June 2014. | | * | | * | * | | * | | ** | | * | | * | * | | 9 |
| Dawe et al. Clinical Lung Cancer. 17 (6) (pp 563-572.e2), 2016. Date of Publication: 01 v 2016. | | * | |  | * | | * | |  | | * | | * | * | | 6 |
| de Decker et al. PLoS ONE 9(5): e98127. doi:10.1371/journal.pone.0098127 | | * | | * | * | |  | | ** | | * | | * | * | | 8 |
| de Martino et al. BJU Int. 2014 May;113(5b):E62-6. doi: 10.1111/bju.12436. Epub 2013 Dec 2. | | * | |  | * | | * | |  | |  | | * |  | | 4 |
| Defor et al. Bone Marrow Transplantation. 45 (5) (pp 933-938), 2010. Date of Publication: May 2010. | | * | |  | * | | * | |  | | * | | * |  | | 5 |
| Dell’Oglio et al. J Natl Compr Canc Netw. 2017 Mar;15(3):327-333. doi: 10.6004/jnccn.2017.0032. | | * | |  | * | |  | |  | | * | | * |  | | 4 |
| Dell’Oglio et al. Annals of Surgical Oncology. ( pagination), 2019. Date of Publication: 2019. | | * | |  | * | | * | |  | | * | | * |  | | 5 |
| Deschuymer et al. Front Oncol. 2018 Jul 23;8:273. doi: 10.3389/fonc.2018.00273. eCollection 2018. | | * | | * | * | |  | | ** | |  | | * |  | | 6 |
| Dessai et al. South Asian Journal of Cancer. 7 (4) (pp 240-243), 2018. Date of Publication: October-December 2018. | |  | |  | * | | * | |  | |  | | * | * | | 4 |
| Dispinzieri et al. Medical Oncology (2018) 35:107 https://doi.org/10.1007/s12032-018-1165-9 | | * | |  | * | | * | |  | |  | | * |  | | 4 |
| Downs-Canner et al. Cancer. 2019 Dec 20. doi: 10.1002/cncr.32668. [Epub ahead of print] | | * | |  | * | |  | |  | | * | | * | * | | 5 |
| Egleston et al. Health Services Research. 50 (4) (pp 1179-1194), 2015. Date of Publication: 01 Aug 2015. | | * | |  | * | |  | |  | | * | | * |  | | 4 |
| Eisenberg et al. J Urol. 2013 Dec;190(6):2005-10. doi: 10.1016/j.juro.2013.06.022. Epub 2013 Jun 14. | | * | |  | * | |  | |  | | * | | * |  | | 4 |
| El Amrani et al. Ann Surg. 2018 v;268(5):854-860. doi: 10.1097/SLA.0000000000002898. | | * | |  | * | | * | |  | |  | | * | * | | 5 |
| Elshaikh et al. American Journal of Clinical Oncology. 40(2):183-188, APR 2017. | | * | |  | * | | * | |  | | * | | * |  | | 5 |
| Englehardt et al. Haematologica. 2016 Sep;101(9):1110-9. doi: 10.3324/haematol.2016.148189. Epub 2016 Jun 16. | |  | |  |  | | * | | ** | |  | | * | * | | 5 |
| Engelhardt et al. Haematologica. 2017 May;102(5):910-921. doi: 10.3324/haematol.2016.162693. Epub 2017 Feb 2. | | * | |  | * | | * | |  | |  | | * |  | | 4 |
| Esposito et al. J Surg Res. 2018 January ; 221: 49–57. doi:10.1016/j.jss.2017.08.008. | | * | |  | * | | * | |  | | * | | * |  | | 5 |
| Faiz et al. Journal of the American College of Surgeons. 210 (4) (pp 390-401), 2010. Date of Publication: April 2010. | | * | | * | * | | * | | ** | | * | | * |  | | 8 |
| Fallahpour et al. CMAJ Open 2017. DOI:10.9778/cmajo.20170030 | | * | |  | * | | * | |  | | * | | * |  | | 5 |
| Farris et al. International Journal of Cancer. 140 (7) (pp 1517-1527), 2017. Date of Publication: 01 Apr 2017. | | * | |  | * | |  | |  | |  | | * |  | | 3 |
| Ficarra et al. BJU International. 124 (4) (pp 621-628), 2019. Date of Publication: 01 Oct 2019. | | * | |  | * | | * | |  | | * | | * | * | | 6 |
| Fieber et al. J Surg Res. 2018 v;231:380-386. doi: 10.1016/j.jss.2018.05.079. Epub 2018 Jun 29. | | * | |  | * | | * | |  | |  | | * | * | | 5 |
| Firat et al. Int J Radiat Oncol Biol Phys 2010; 78: 1394–1399. | |  | |  | * | | * | |  | | * | | * | * | | 5 |
| Froehner et al. European Urology. 69 (5) (pp 764-766), 2016. Date of Publication: 01 May 2016. | | * | |  | * | | * | |  | |  | | * |  | | 4 |
| Galsky et al. JCO Clin Cancer Inform. 2017 v;1:1-12. doi: 10.1200/CCI.17.00116. | | * | |  | * | | * | |  | |  | | * |  | | 4 |
| Gannon et al. J Geriatr Oncol. 2020 Jan 29. pii: S1879-4068(19)30494-1. doi: 10.1016/j.jgo.2020.01.005. [Epub ahead of print] | | * | |  | * | |  | |  | | * | | * | * | | 5 |
| Ganti et al. Am J Clin Oncol. 2011 Dec;34(6):593-6. doi: 10.1097/COC.0b013e3181fe445b. | | * | |  | * | | * | |  | | * | | * | * | | 6 |
| Gordon et al. Cancer. 124 (15) (pp 3192-3200), 2018. Date of Publication: August 2018. | | * | |  | * | | * | |  | |  | | * | * | | 5 |
| Graboyes et al. JAMA Otolaryngol Head Neck Surg. 2016 May 1;142(5):430-7. doi: 10.1001/jamaoto.2015.3595. | | * | |  | * | | * | |  | | * | | * |  | | 5 |
| Graf et al. Biology of Blood and Marrow Transplantation. 22 (9) (pp 1582-1587), 2016. Date of Publication: 01 Sep 2016. | | * | |  | * | | * | |  | |  | | * |  | | 4 |
| Graham-Steed et al. Journal of Geriatric Oncology. 5 (4) (pp 343-351), 2014. Date of Publication: 01 Oct 2014. | | * | |  | * | |  | |  | | * | | * | * | | 5 |
| Gray et al. PLoS Med. 2019 Dec 31;16(12):e1003006. doi: 10.1371/journal.pmed.1003006. eCollection 2019 Dec. | | * | | * | * | | * | | ** | | * | | * | * | | 9 |
| Guevara et al. European Journal of Cancer Prevention 2017, 26:S215–S222 | | * | | * | * | |  | | ** | | * | | * |  | | 7 |
| Gupta et al. Clin Sarcoma Res. 2016 Jul 19;6:12. doi: 10.1186/s13569-016-0051-5. eCollection 2016. | | * | |  | * | | * | |  | | * | | * |  | | 5 |
| Habbous et al. Oral Oncol. 2013 May;49(5):407-12. doi: 10.1016/j.oraloncology.2012.11.010. Epub 2013 Jan 4. | | * | |  | * | |  | |  | | * | | * | * | | 5 |
| Hamstra et al. Int J Radiat Oncol Biol Phys. 2013 Apr 1;85(5):1246-53. doi: 10.1016/j.ijrobp.2012.10.042. Epub 2012 Dec 19. | | * | | * | * | |  | | ** | | * | | * |  | | 7 |
| Haque et al. Radiotherapy and Oncology. 129 (2) (pp 264-269), 2018. Date of Publication: vember 2018. | | * | | * | * | | * | | ** | | * | | * |  | | 8 |
| Hashmi et al. Leuk Res. 2013 Sep;37(9):1052-6. doi: 10.1016/j.leukres.2013.06.013. Epub 2013 Jul 3. | | * | |  | * | | * | |  | | * | | * |  | | 5 |
| Hermet et al. Journal of Geriatric Oncology 2015;6;346-352. | | * | |  | * | | * | |  | | * | | * |  | | 5 |
| Herskovic et al. Clinical Breast Cancer, Vol. 18, . 5, 418-31 | | * | | * | * | | * | | ** | | * | | * |  | | 8 |
| Huang et al. Chin J Cancer. 2017 Feb 27;36(1):23. doi: 10.1186/s40880-017-0192-9. | | * | |  | * | | * | |  | | * | | * |  | | 5 |
| Huang et al. Radiother Oncol. 2018 v;129(2):389-395. doi: 10.1016/j.radonc.2018.09.004. Epub 2018 Sep 27. | | * | |  | * | | * | |  | | * | | * |  | | 5 |
| Hung et al. J Ethpharmacol. 2017 Mar 6;199:168-174. doi: 10.1016/j.jep.2017.02.004. Epub 2017 Feb 3. | | * | | * | * | | * | | ** | | * | | * |  | | 8 |
| Irisa et al. Med Oncol. 2012 Mar;29(1):185-92. doi: 10.1007/s12032-010-9764-0. Epub 2010 Dec 7. | | * | |  | * | | * | |  | |  | | * |  | | 4 |
| Ito et al. Dig Surg. 2017;34(1):78-85. doi: 10.1159/000446928. Epub 2016 Jul 28. | |  | |  | * | |  | |  | | * | | * | * | | 4 |
| Iwamoto et al. Int J Clin Oncol. 2016 Aug;21(4):756-763. doi: 10.1007/s10147-016-0950-8. Epub 2016 Jan 20. | |  | | * | * | | * | | ** | | * | | * | * | | 8 |
| Jiralerspong et al. Annals of Oncology 24: 2506–2514, 2013 doi:10.1093/annc/mdt224 | | * | |  | * | | * | |  | | * | | * |  | | 5 |
| Jitawatanarat et al. J Breast Cancer. 2014 Dec;17(4):356-62. doi: 10.4048/jbc.2014.17.4.356. Epub 2014 Dec 26. | | * | | * | * | | * | | ** | | * | | * | * | | 9 |
| Kaesmann et al. Lung. 194 (2) (pp 295-298), 2016. Date of Publication: 01 Apr 2016. | |  | |  |  | | * | |  | |  | | * |  | | 2 |
| Kollogjeri et al. Journal of the American College of Surgeons. 219 (2) (pp 245-255), 2014. Date of Publication: August 2014. | | * | |  | * | | * | |  | |  | | * |  | | 4 |
| Kang et al. International Journal of Radiation Oncology Biology Physics. 106 (1) (pp 90-99), 2020. Date of Publication: 1 January 2020. | | * | |  | * | | * | |  | |  | | * |  | | 4 |
| Kappa et al. J Urol. 2017 Sep;198(3):560-566. doi: 10.1016/j.juro.2017.04.018. Epub 2017 Apr 8. | | * | |  |  | |  | |  | | * | | * |  | | 3 |
| Kaufmann et al. Support Care Cancer. 2015 Jul;23(7):1883-92. doi: 10.1007/s00520-014-2546-z. Epub 2014 Dec 6. | |  | |  | * | |  | |  | |  | | * |  | | 2 |
| Killelea et al. J Clin Oncol 33:4267-4275. | | * | | * | * | |  | | ** | | * | | * |  | | 7 |
| Kim et al. Clin Cancer Res. 2019 Aug 15;25(16):5143-5155. doi: 10.1158/1078-0432.CCR-18-3988. Epub 2019 Jun 28. | | * | |  | * | | * | |  | |  | | * |  | | 4 |
| Kleber et al. Clinical lymphoma, myeloma & leukemia. 13(5):541-51, 2013 Oct. | | * | |  | * | | * | |  | |  | | * | * | | 5 |
| KleinJan et al. J Robot Surg. 2019 Jun;13(3):455-462. doi: 10.1007/s11701-018-0867-5. Epub 2018 Sep 3. | | * | |  | * | | * | |  | |  | | * |  | | 4 |
| Klement et al. J Thorac Oncol. 2016 Jul;11(7):1132-9. doi: 10.1016/j.jtho.2016.03.016. Epub 2016 Apr 7. | | * | |  | * | | * | |  | | * | | * | * | | 6 |
| Kluth et al. BJU Int. 2014 Jun;113(6):911-7. doi: 10.1111/bju.12369. | |  | |  | * | | * | |  | | * | | * | * | | 5 |
| Kober et al. Biological research for nursing. 18 (4) (pp 370-385), 2016. Date of Publication: 01 Jul 2016. | |  | | * | * | |  | | ** | |  | | * |  | | 5 |
| Kocher et al. Urol Oncol. 2018 Apr;36(4):156.e17-156.e22. doi: 10.1016/j.urolonc.2017.12.004. Epub 2017 Dec 21. | | * | | * | * | | * | | ** | | * | | * |  | | 8 |
| Kondalsamy-Chennakesavan et al. Eur J Cancer. 2012 Sep;48(14):2155-62. doi: 10.1016/j.ejca.2012.03.013. Epub 2012 Apr 12. | | * | | * | * | | * | | ** | | * | | * | * | | 9 |
| Konglund et al. Acta Neurochir (Wien). 2013 Dec;155(12):2263-71; discussion 2271. doi: 10.1007/s00701-013-1872-0. Epub 2013 Sep 13. | |  | |  | * | | * | |  | | * | | * |  | | 4 |
| Kos et al. Wiener Klinische Wochenschrift. 126 (1-2) (pp 36-41), 2014. Date of Publication: January 2014. | | * | |  | * | | * | |  | | * | | * |  | | 5 |
| Kozower et al. Ann Thorac Surg. 2012 May;93(5):1690-6; discussion 1696-8. doi: 10.1016/j.athoracsur.2012.01.111. | | * | |  | * | | * | |  | |  | | * |  | | 4 |
| Kozower et al. Ann Surg. 2011 Dec;254(6):1032-7. doi: 10.1097/SLA.0b013e31821d4bdd. | | * | |  | * | | * | |  | |  | | * |  | | 4 |
| Kutikov et al. J Clin Oncol 28(2):311–317 | | * | | * | * | | * | | ** | | * | | * |  | | 8 |
| Kwon et al. Bone Marrow Transplant. 2017 Aug;52(8):1138-1143. doi: 10.1038/bmt.2017.36. Epub 2017 Mar 27. | | * | | * | * | | * | | ** | |  | | * |  | | 7 |
| Lakomkin et al. Spine (Phila Pa 1976). 2019 Jul 1;44(13):E782-E787. doi: 10.1097/BRS.0000000000002970. | | * | |  | * | | * | |  | | * | | * |  | | 5 |
| Lange et al. Oncologist. 24 (1) (pp 62-68), 2019. Date of Publication: January 2019. | | * | |  | * | | * | | ** | |  | | * | * | | 7 |
| Lange et al. Oncologist. 21 (11) (pp 1337-1348), 2016. Date of Publication: vember 2016. | |  | |  |  | | * | | ** | |  | | * | * | | 5 |
| Large et al. Urology. 2013 Jan;81(1):123-8. doi: 10.1016/j.urology.2012.07.086. Epub 2012 v 13. | |  | |  | * | |  | |  | | * | | * | * | | 4 |
| Lascano et al. Urol Oncol. 2015 Oct;33(10):426.e1-12. doi: 10.1016/j.urolonc.2015.06.002. Epub 2015 Jul 9. | | * | | * | * | | * | | ** | |  | | * |  | | 7 |
| Lee et al. Auris Nasus Larynx. 43 (3) (pp 322-329), 2016. Date of Publication: 01 Jun 2016. | | * | |  | * | | * | |  | | * | | * | * | | 6 |
| Lewis et al. Annals of Surgical Oncology. 26 (5) (pp 1351-1357), 2019. Date of Publication: 15 May 2019. | | * | | * | * | | * | | ** | | * | | * |  | | 8 |
| Li et al. Cancer Med. 2017 v;6(11):2586-2594. doi: 10.1002/cam4.1224. Epub 2017 Oct 4. | | * | |  | * | | * | |  | | * | | * | * | | 6 |
| Lichtensztajn et al. Clinical Epidemiology. 9 (pp 601-609), 2017. Date of Publication: 20 v 2017. | | * | |  | * | | * | |  | | * | | * | * | | 6 |
| Lieffers et al. Cancer 117, 1957–1965 (2011) | | * | |  | * | | * | |  | |  | | * | * | | 5 |
| Lin et al. BMC Surg. 2019 May 27;19(1):53. doi: 10.1186/s12893-019-0513-9. | | * | | * | * | | * | | ** | | * | | * |  | | 8 |
| Lindviksmoen et al. Cancer Nursing. 36 (6) (pp E34-E43), 2013. Date of Publication: vember-December 2013. | | * | |  | * | | * | |  | |  | | * |  | | 4 |
| Liu et al. Oncologist. 23 (6) (pp 722-729), 2018. Date of Publication: June 2018. | |  | |  | * | | * | |  | | * | | * | * | | 5 |
| Liu et al. Blood. 134 (4) (pp 374-382), 2019. Date of Publication: 25 Jul 2019. | | * | |  | * | |  | |  | | * | | * | * | | 5 |
| Lombardi et al. Cancers. 11 (10) ( pagination), 2019. Article Number: 1509. Date of Publication: October 2019. | | * | |  | * | | * | |  | | * | | * |  | | 5 |
| Longo et al. BJU Int. 2016 Oct;118(4):521-6. doi: 10.1111/bju.13462. Epub 2016 Apr 4. | | * | | * | * | | * | | ** | | * | | * | * | | 9 |
| Louie et al. International Journal of Radiation Oncology Biology Physics. 93 (1) (pp 82-90), 2015. Date of Publication: 01 Sep 2015. | | * | |  | * | | * | |  | | * | | * |  | | 5 |
| Luchtenborg et al. Thorax. 2018 Apr;73(4):339-349. doi: 10.1136/thoraxjnl-2017-210362. Epub 2017 Oct 27. | | * | |  | * | |  | | ** | | * | | * |  | | 6 |
| Margalit et al. International Journal of Radiation Oncology Biology Physics. 81 (5) (pp e735-e741), 2011. Date of Publication: 01 Dec 2011. | |  | |  | * | |  | |  | | * | | * |  | | 3 |
| Maringe et al. PLoS One. 2017 Mar 6;12(3):e0172814. doi: 10.1371/journal.pone.0172814. eCollection 2017. | | * | |  | * | |  | |  | | * | | * |  | | 4 |
| Mayr et al. Eur Urol. 2012 Oct;62(4):662-70. doi: 10.1016/j.eururo.2012.03.057. Epub 2012 Apr 12. | | * | |  | * | | * | | ** | | * | | * |  | | 7 |
| Mayr et al. BJU International 2012;110:E222-E227. | | * | |  | * | | * | | ** | | * | | * |  | | 7 |
| Mayr et al. Journal of Cachexia, Sarcopenia and Muscle 2018; 9: 505–513 | | * | | * | * | | * | | ** | |  | | * |  | | 7 |
| McCarthy et al. BMC Cancer. 2018 Sep 14;18(1):892. doi: 10.1186/s12885-018-4807-6. | | * | |  | * | |  | |  | | * | | * | * | | 5 |
| Mehta et al. Cancer. 124 (9) (pp 2018-2025), 2018. Date of Publication: 01 May 2018. | | * | | * | * | | * | | * | | * | | * |  | | 7 |
| Michelis et al. Bone Marrow Transplant. 2015 v;50(11):1405-10. doi: 10.1038/bmt.2015.165. Epub 2015 Jul 13. | | * | |  | * | | * | |  | | * | | * |  | | 5 |
| Milne et al. British Journal of Oral and Maxillofacial Surgery. 57 (3) (pp 214-218), 2019. Date of Publication: April 2019. | | * | |  | * | | * | |  | | * | | * |  | | 5 |
| Mischinger et al. Eur J Surg Oncol. 2018 Jan;44(1):178-184. doi: 10.1016/j.ejso.2017.10.208. Epub 2017 Oct 20. | |  | | * | * | | * | | ** | |  | | * | * | | 7 |
| Mo et al. Am J Hematol. 2013 Jun;88(6):497-502. doi: 10.1002/ajh.23443. Epub 2013 May 8. | | * | |  | * | | * | |  | | * | | * |  | | 5 |
| Montero et al. Cancer 2014 Jan 15;120(2):214-21. doi: 10.1002/cncr.28407. Epub 2013 Oct 25. | | * | |  | * | | * | |  | | * | | * |  | | 5 |
| Morgan et al. Urology. 80 (3) (pp 632-638), 2012. Date of Publication: September 2012. | | * | | * | * | | * | | ** | | * | | * |  | | 8 |
| Morgan et al. J Urol. 2011 Sep;186(3):829-34. doi: 10.1016/j.juro.2011.04.089. Epub 2011 Jul 23. | | * | |  | * | | * | |  | |  | | * | * | | 5 |
| Muffly et al. Haematologica, 99, 1373–1379. | | * | | * | * | | * | | ** | |  | | * |  | | 7 |
| Nakajo et al. J Gastroenterol. 2019 Oct;54(10):871-880. doi: 10.1007/s00535-019-01583-9. Epub 2019 May 4. | | * | | * | * | | * | | ** | | * | | * | * | | 9 |
| Nakaya et al. Biology of Blood and Marrow Transplantation. 20 (10) (pp 1553-1559), 2014. Date of Publication: 01 Oct 2014. | | * | |  | * | | * | |  | |  | | * |  | | 4 |
| Naqvi et al. Blood. 130(Suppl. 1):4258, DEC 7 2017. | | * | |  | * | |  | |  | | * | | * |  | | 4 |
| Naumann et al. Anticancer Res. 2013 Apr;33(4):1717-20. | | * | |  | * | |  | |  | | * | | * | * | | 5 |
| Nepple et al. European Urology. 64 (3) (pp 372-378), 2013. Date of Publication: September 2013. | | * | | * | * | | * | | ** | | * | | * |  | | 8 |
| Noer et al. Gynecologic Oncology. 141 (3) (pp 471-478), 2016. Date of Publication: June 2016. | | * | |  | * | | * | |  | | * | | * | * | | 6 |
| Novara et al. Journal of Urology. 184 (3) (pp 1028-1033), 2010. Date of Publication: September 2010. | | * | |  | * | | * | |  | |  | | * |  | | 4 |
| Osthus et al. European Archives of Oto-Rhi-Laryngology. 270 (5) (pp 1721-1728), 2013. Date of Publication: May 2013. | | * | |  | * | | * | |  | |  | | * |  | | 4 |
| Parakh et al. Journal of Geriatric Oncology 2015;6:387-394. | | * | | * | * | |  | | ** | | * | | * |  | | 7 |
| Pule et al. Journal of Evaluation in Clinical Practice. ( pagination), 2019. Date of Publication: 2019. | | * | |  | * | | * | |  | | * | | * | * | | 6 |
| Quintana et al. PLoS One. 2018 Jun 28;13(6):e0199894. doi: 10.1371/journal.pone.0199894. eCollection 2018. | | * | |  | * | | * | |  | | * | | * |  | | 5 |
| Quintana et al. Colorectal Dis. 2018 Aug;20(8):676-687. doi: 10.1111/codi.14250. Epub 2018 May 30. | | * | |  | * | | * | |  | | * | | * | * | | 6 |
| Ragulin-Coyne et al. Surgery. 2012 Sep;152(3 Suppl 1):S120-7. doi: 10.1016/j.surg.2012.05.018. Epub 2012 Jul 3. | | * | |  | * | | * | |  | | * | | * |  | | 5 |
| Raimondi et al. Blood. 120 (6) (pp 1327-1333), 2012. Date of Publication: 09 Aug 2012. | | * | |  | * | | * | |  | | * | | * | * | | 6 |
| Raman t al. BJU International. 119 (2) (pp 268-275), 2017. Date of Publication: 01 Feb 2017. | | * | |  | * | | * | |  | | * | | * | * | | 6 |
| Ray-Zack et al. Urol Oncol. 2019 v;37(11):837-843. doi: 10.1016/j.urolonc.2018.10.024. Epub 2018 v 13. | | * | |  | * | | * | |  | | * | | * | * | | 6 |
| Reeder-Hayes et al. Journal of Clinical Oncology. 34 (17) (pp 2003-2009), 2016. Date of Publication: 10 Jun 2016. | | * | |  | * | | * | |  | | * | | * |  | | 5 |
| Reeve et al. BJU International. Part B. 114 (6) (pp E74-E81), 2014. Date of Publication: 01 Dec 2014. | | * | |  | * | | * | | ** | |  | | * |  | | 6 |
| Reha et al. European Journal of Surgical Oncology. 44 (1) (pp 148-156), 2018. Date of Publication: January 2018. | | * | | * | * | | * | | ** | |  | | * |  | | 7 |
| Reyes et al. Urol Oncol. 2013 Oct;31(7):1292-7. doi: 10.1016/j.urolonc.2011.09.006. Epub 2012 Apr 21. | |  | |  | * | | * | |  | | * | | * | * | | 5 |
| Rietbergen et al. Br J Cancer. 2015 May 26;112(11):1733-6. doi: 10.1038/bjc.2015.139. Epub 2015 May 7. | | * | |  | * | | * | |  | |  | | * |  | | 4 |
| Rios-Velazquez et al. Radiother Oncol. 2014 Dec;113(3):324-30. doi: 10.1016/j.radonc.2014.09.005. Epub 2014 Oct 24. | | * | |  | * | | * | |  | |  | | * |  | | 4 |
| Ritch et al. J Urol. 2014 v;192(5):1360-4. doi: 10.1016/j.juro.2014.06.004. Epub 2014 Jun 10. | | * | |  | * | | * | |  | | * | | * | * | | 6 |
| Riviere et al. JCO Clinical Cancer Informatics. (3) ( pagination), 2019. Date of Publication: 2019. | | * | |  | * | | * | |  | | * | | * | * | | 6 |
| Roseland et al. Breast Cancer Research and Treatment. 165 (1) (pp 163-168), 2017. Date of Publication: 01 Aug 2017. | | * | | * | * | |  | | ** | | * | | * |  | | 7 |
| Roxburgh et al. Int J Colorectal Dis. 2011 Apr;26(4):483-92. doi: 10.1007/s00384-010-1120-5. Epub 2011 Jan 7. | | * | |  | * | |  | |  | | * | | * | * | | 5 |
| Rush et al. Can Urol Assoc J. 2015 May-Jun;9(5-6):179-87. doi: 10.5489/cuaj.2618. | | * | | * | * | |  | | ** | |  | | * |  | | 6 |
| Rusthoven et al. Annals of Oncology. 27 (5) (pp 818-827), 2016. Article Number: mdw018. Date of Publication: 01 May 2016. | | * | | * | * | | * | | ** | | * | | * |  | | 8 |
| Saeed et al. Hematol Oncol Stem Cell Ther. 2018 Jun;11(2):90-95. doi: 10.1016/j.hemonc.2017.12.002. Epub 2018 Feb 3. | |  | |  | * | | * | |  | | * | | * |  | | 4 |
| Salit et al. Biol Blood Marrow Transplant. 2017 Apr;23(4):654-658. doi: 10.1016/j.bbmt.2017.01.084. Epub 2017 Feb 9. | | * | |  | * | | * | |  | |  | | * |  | | 4 |
| Saluk et al. Current Urology. 12 (1) (pp 20-26), 2018. Date of Publication: 01 Oct 2018. | | * | |  | * | | * | |  | |  | | * |  | | 4 |
| Sandler et al. Ann Surg Oncol 23:638-645, 2016 | | * | |  | * | | * | |  | |  | | * |  | | 4 |
| Sandri et al. Journal of Thoracic Disease. 8 (8) (pp 2121-2127), 2016. Date of Publication: 2016. | | * | |  | * | | * | |  | | * | | * |  | | 5 |
| Sarfati et al. Med Care. 2014 Jul;52(7):586-93. doi: 10.1097/MLR.0000000000000149. | | * | | * | * | |  | | ** | | * | | * | * | | 8 |
| Sarfati et al. Journal of Clinical Epidemiology. 67 (5) (pp 586-595), 2014. Date of Publication: May 2014. | | * | | * | * | |  | | ** | | * | | * |  | | 7 |
| Schandl et al. BMJ Open. 2016 Aug 26;6(8):e012624. doi: 10.1136/bmjopen-2016-012624. | | * | |  | * | |  | |  | |  | | * |  | | 3 |
| Sena et al. JMIR Cancer. 2019 Sep 26;5(2):e12163. doi: 10.2196/12163. | | * | |  | * | | * | |  | |  | | * |  | | 4 |
| Shi et al. J Gastrointest Surg. 2012 v;16(11):2126-31. doi: 10.1007/s11605-012-1986-3. Epub 2012 Aug 10. | | * | |  | * | | * | |  | |  | | * |  | | 4 |
| Shouval et al. Blood Advances. 3 (12) (pp 1881-1890), 2019. Date of Publication: 2019. | | * | |  | * | | * | |  | |  | | * | * | | 5 |
| Simon et al. Eur J Radiol. 2012 Dec;81(12):4167-72. doi: 10.1016/j.ejrad.2012.06.007. Epub 2012 Jul 24. | | * | |  | * | | * | |  | | * | | * |  | | 5 |
| Simons et al. Cancer. 116 (7) (pp 1733-1738), 2010. Date of Publication: 01 Apr 2010. | | * | |  | * | | * | |  | | * | | * |  | | 5 |
| Slieker et al. Head Neck. 2019 Oct;41(10):3584-3593. doi: 10.1002/hed.25879. Epub 2019 Jul 26. | | * | |  | * | | * | |  | |  | | * |  | | 4 |
| Sorror et al. Biology of Blood and Marrow Transplantation. 21 (8) (pp 1479-1487), 2015. Date of Publication: 01 Aug 2015. | | * | | * | * | | * | | ** | | * | | * |  | | 8 |
| Sorror et al. Journal of Clinical Oncology. 32 (29) (pp 3249-3256), 2014. Date of Publication: 10 Oct 2014. | | * | |  | * | | * | |  | |  | | * | * | | 5 |
| Sorror et al. JAMA Oncol. 2017 Dec 1;3(12):1675-1682. doi: 10.1001/jamaoncol.2017.2714. | | * | |  | * | | * | |  | | * | | * | * | | 6 |
| Statler et al. Sci Rep. 2019 Dec 2;9(1):18126. doi: 10.1038/s41598-019-54402-9. | | * | |  | * | | * | |  | |  | | * |  | | 4 |
| Suardi et al. European Urology. 65 (3) (pp 546-551), 2014. Date of Publication: March 2014. | | * | | * | * | | * | | ** | |  | | * |  | | 7 |
| Takada et al. Anticancer Research. 39 (6) (pp 2941-2950), 2019. Date of Publication: 2019. | | * | |  | * | | * | |  | | * | | * |  | | 5 |
| Takenaka et al. Sci Rep. 2017 Aug 4;7(1):7297. doi: 10.1038/s41598-017-07752-1. | | * | |  | * | | * | |  | |  | | * |  | | 4 |
| Tan et al. J Postgrad Med. 2014 Jul-Sep;60(3):248-53. doi: 10.4103/0022-3859.138726. | | * | |  | * | | * | |  | |  | | * |  | | 4 |
| Te Riele et al. Oral Oncol. 2018 Mar;78:25-30. doi: 10.1016/j.oraloncology.2018.01.001. Epub 2018 Jan 11. | | * | |  | * | | * | |  | |  | | * | * | | 5 |
| Terwey et al. Haematologica, 95, 810–818. | | * | |  | * | | * | |  | |  | | * |  | | 4 |
| Thanarajasingam et al. Biology of Blood and Marrow Transplantation. 19 (12) (pp 1713-1718), 2013. Date of Publication: December 2013. | | * | |  | * | | * | |  | | * | | * |  | | 5 |
| Tinguely et al. Eur J Surg Oncol. 2019 Dec 2:S0748-7983(19)31495-7. doi: 10.1016/j.ejso.2019.12.002. Online ahead of print. | | * | | * | * | | * | | ** | |  | | * |  | | 7 |
| Tokunaga et al. Leukemia & Lymphoma. 58(1):37-44, 2017 01. | | * | |  | * | | * | |  | |  | | * |  | | 4 |
| Torlen et al. Biology of Blood and Marrow Transplantation. 23 (4) (pp 677-683), 2017. Date of Publication: 01 Apr 2017. | | * | |  | * | | * | |  | |  | | * |  | | 4 |
| Toxopeus et al. Radiother Oncol. 2015 Jun;115(3):392-8. doi: 10.1016/j.radonc.2015.04.028. Epub 2015 Jun 23. | | * | |  | * | | * | |  | |  | | * |  | | 4 |
| Tran et al. Radiother Oncol. 2013 Jun;107(3):366-71. doi: 10.1016/j.radonc.2013.05.003. Epub 2013 May 29. | | * | | * | * | | * | | ** | | * | | * | * | | 9 |
| Tumbarello et al. PLoS ONE. 7 (12) ( pagination), 2012. Article Number: e51612. Date of Publication: 14 Dec 2012. | | * | |  | * | | * | |  | |  | | * |  | | 4 |
| Uhlig et al. Radiology. 288 (3) (pp 889-897), 2018. Date of Publication: September 2018. | | * | | * | * | | * | | ** | | * | | * | * | | 9 |
| Vaz-Luis et al. J Clin Oncol 32:927-934. DOI: 10.1200/JCO.2013.51.1261 | | * | |  | * | | * | |  | | * | | * | * | | 6 |
| Veeraputhiran et al. Biology of Blood and Marrow Transplantation. 23 (10) (pp 1744-1748), 2017. Date of Publication: October 2017. | | * | |  | * | | * | |  | |  | | * |  | | 4 |
| Verslius et al. Leukemia. 29 (1) (pp 51-57), 2015. Date of Publication: 10 Jan 2015. | | * | |  | * | | * | |  | |  | | * | * | | 5 |
| Vetterlein et al. Eur Urol. 2020 Jan;77(1):55-65. doi: 10.1016/j.eururo.2019.08.011. Epub 2019 Aug 29. | | * | |  | * | | * | |  | | * | | * |  | | 5 |
| Vitzthum et al. JCO Clin Cancer Inform. 2018 Dec;2:1-9. doi: 10.1200/CCI.18.00082. | |  | |  |  | |  | |  | |  | | * | * | | 2 |
| Wagner et al. Eur J Surg Oncol 2018;44:658–63. | | * | |  | * | | * | |  | |  | | * |  | | 4 |
| Wahlgreen et al. Int J Radiat Oncol Biol Phys. 2011 v 15;81(4):997-1004. doi: 10.1016/j.ijrobp.2010.07.014. Epub 2010 Oct 6. | | * | |  | * | |  | |  | |  | | * | * | | 4 |
| Wang et al. J Clin Oncol 2012; 30: 1447–1455. | | * | |  | * | |  | |  | | * | | * | * | | 5 |
| Weiner et al. Journal of Urology. 193 (1) (pp 95-102), 2015. Date of Publication: January 2015. | | * | |  | * | | * | |  | | * | | * |  | | 5 |
| Weingart et al. Cancer Medicine. ( pagination), 2020. Date of Publication: 2020. | | * | |  | * | |  | |  | | * | | * | * | | 5 |
| Wen et al. Journal of Cancer. 10 (23) (pp 5614-5621), 2019. Date of Publication: 2019. | | * | | * | * | | * | | ** | |  | | * |  | | 7 |
| Williams et al. Journal of Oncology Practice. 15 (5) (pp E399-E409), 2019. Date of Publication: 01 May 2019. | | * | |  | * | |  | |  | | * | | * |  | | 4 |
| Woldu et al. Cancer. 2019 Jan 15;125(2):223-231. doi: 10.1002/cncr.31799. Epub 2018 Oct 6. | | * | |  | * | | * | |  | | * | | * |  | | 5 |
| Wong et al. Journal of Oncology Practice. 14 (10) (pp e631-e643), 2018. Date of Publication: 01 Oct 2018. | | * | | * | * | |  | | ** | | * | | * | * | | 8 |
| Wood et al. Pediatr Blood Cancer. 2011 Sep;57(3):499-505. doi: 10.1002/pbc.23057. Epub 2011 Mar 7. | |  | |  | * | | * | |  | | * | | * |  | | 4 |
| Wright et al. Fatigue: Biomedicine, Health and Behavior. 5 (3) (pp 131-144), 2017. Date of Publication: 03 Jul 2017. | | * | |  | * | |  | |  | |  | | * |  | | 3 |
| Wright et al. Cancer Nursing. 42 (5) (pp 355-364), 2019. Date of Publication: 01 Sep 2019. | | * | |  | * | |  | |  | |  | | * |  | | 3 |
| Wurtzen et al. Psycho-Oncology. 22 (5) (pp 1180-1185), 2013. Date of Publication: May 2013. | | * | | * | * | |  | | ** | | * | | * |  | | 7 |
| Xu et al. Journal of the American Medical Informatics Association. 22 (1) (pp 179-191), 2015. Date of Publication: 2015. | | * | | * | * | | * | | ** | |  | | * |  | | 7 |
| Yadav et al. DOI: 10.1002/cncr.32472, | | * | |  | * | | * | |  | |  | | * |  | | 4 |
| Yamamoto et al. Am J Hematol. 2014 Sep;89(9):E138-41. doi: 10.1002/ajh.23764. Epub 2014 Jun 20. | |  | |  | * | | * | |  | |  | | * |  | | 3 |
| Yang et al. European Journal of Cardio-thoracic Surgery. 53 (1) (pp 235-240), 2018. Date of Publication: January 2018. | | * | |  | * | | * | |  | | * | | * | * | | 6 |
| Yang et al. PLoS One. 2015 Jan 24;10(1):e0117323. doi: 10.1371/journal.pone.0117323. eCollection 2015. | | * | |  | * | | * | |  | | * | | * | * | | 6 |
| Yusuf et al. Curr Med Res Opin. 2016 Dec;32(12):1989-1996. doi: 10.1080/03007995.2016.1226166. Epub 2016 Sep 16. | | * | |  | * | | * | |  | | * | | * |  | | 5 |
| Zancan et al. European journal of physical and rehabilitation medicine. 55 (6) (pp 735-742), 2019. Date of Publication: 01 Dec 2019. | | * | |  | * | | * | |  | | * | | * |  | | 5 |
| Zhang et al. Breast Cancer Res Treat. 2018 May ; 169(1): 175–187. doi:10.1007/s10549-017-4646-1. | | * | | * | * | | * | | ** | | * | | * |  | | 8 |
| Newcastle Ottawa Criteria for Risk of Bias: Case-Control Studies | | | | | | | | | | | | | | | | |
| Citation | | **Is the case definition adequate?** | | **Representativeness of the cases** | **Selection of Controls** | | **Definition of Controls** | | **Comparability of cases and controls on the basis of the design or analysis** | | **Ascertainment of exposure** | | **Same method of ascertainment for cases and controls** | **Non-Response rate** | | **Total *** |
| Thygesen et al. British Journal of Clinical Pharmacology. 83 (11) (pp 2517-2527), 2017. Date of Publication: November 2017. | |  | | * | * | | * | | ** | | * | | * |  | | 7 |
| Vinograd et al. Clin Microbiol Infect. 2014 Sep;20(9):899-905. doi: 10.1111/1469-0691.12625. Epub 2014 Apr 10. | |  | | * |  | | * | | ** | | * | | * |  | | 6 |
| Cochrane Criteria for Risk of Bias: Clinical Trials | | | | | | | | | | | | | | | | |
| Citation | **Random Sequence Generation** | | **Allocation Concealment** | | | **Blinding of participants and personnel** | | **Blinding of outcome assessment** | | **Incomplete outcome data** | | **Selective reporting** | | | **Overall (median^a^)** | |
| Asmis et al. Annals of Oncology. 22 (1) (pp 118-126), 2011. Date of Publication: 2011. | Low Risk | | Unclear Risk | | | Unclear Risk | | Unclear Risk | | Unclear Risk | | Low Risk | | | Unclear Risk | |
| Jeppesen et al. J Geriatr Oncol. 2018 Nov;9(6):575-582. doi: 10.1016/j.jgo.2018.05.009. Epub 2018 Jun 3. | Low Risk | | Unclear Risk | | | Unclear Risk | | Unclear Risk | | High Risk | | Low Risk | | | Unclear Risk | |
| Merli et al. Leuk Lymphoma. 2014 Jan;55(1):38-43. doi: 10.3109/10428194.2013.788176. Epub 2013 Apr 30. | Low Risk | | Unclear Risk | | | Unclear Risk | | Unclear Risk | | Low Risk | | Low Risk | | | Unclear Risk | |
| Parekh et al. J Urol. 2013 Feb;189(2):474-9. doi: 10.1016/j.juro.2012.09.077. Epub 2012 Sep 24. | Low Risk | | Low Risk | | | High Risk | | Unclear Risk | | Low Risk | | Low Risk | | | Low Risk | |
| Saussele et al. Blood. 2015 Jul 2;126(1):42-9. doi: 10.1182/blood-2015-01-617993. Epub 2015 Apr 27. | Low Risk | | Unclear Risk | | | Unclear Risk | | Unclear Risk | | High Risk | | Low Risk | | | Unclear Risk | |
| von Minckwitz et al. DOI: 10.1002/cncr.29506 | Low Risk | | High Risk | | | High Risk | | High Risk | | High Risk | | Low Risk | | | High Risk | |

^a^If median is between two different risk levels, overall column indicates the worse risk of bias
